# Conditional inference in *cis*‐Mendelian randomization using weak genetic factors

**DOI:** 10.1111/biom.13888

**Published:** 2023-06-19

**Authors:** Ashish Patel, Dipender Gill, Paul Newcombe, Stephen Burgess

**Affiliations:** ^1^ MRC Biostatistics Unit University of Cambridge Cambridge UK; ^2^ Department of Epidemiology and Biostatistics Imperial College London London UK; ^3^ Chief Scientific Advisor Office Research and Early Development Novo Nordisk, Copenhagen Denmark; ^4^ Cardiovascular Epidemiology Unit University of Cambridge Cambridge UK

**Keywords:** approximate factor models, *cis*‐Mendelian randomization, weak instruments

## Abstract

Mendelian randomization (MR) is a widely used method to estimate the causal effect of an exposure on an outcome by using genetic variants as instrumental variables. MR analyses that use variants from only a single genetic region (*cis*‐MR) encoding the protein target of a drug are able to provide supporting evidence for drug target validation. This paper proposes methods for *cis*‐MR inference that use many correlated variants to make robust inferences even in situations, where those variants have only weak effects on the exposure. In particular, we exploit the highly structured nature of genetic correlations in single gene regions to reduce the dimension of genetic variants using factor analysis. These genetic factors are then used as instrumental variables to construct tests for the causal effect of interest. Since these factors may often be weakly associated with the exposure, size distortions of standard *t*‐tests can be severe. Therefore, we consider two approaches based on conditional testing. First, we extend results of commonly‐used identification‐robust tests for the setting where estimated factors are used as instruments. Second, we propose a test which appropriately adjusts for first‐stage screening of genetic factors based on their relevance. Our empirical results provide genetic evidence to validate cholesterol‐lowering drug targets aimed at preventing coronary heart disease.

## 1 INTRODUCTION

Mendelian randomization (MR) is a widely‐used method to estimate the causal effect of an exposure on an outcome by using genetic variants as instrumental variables. An emerging area of clinical research concerns MR studies that use genetic variants from only single genetic regions of pharmacological interest (*cis*‐MR). Compared with polygenic MR where variants may be chosen from multiple gene regions, *cis*‐MR is often used when the exposure of interest is regulated by a specific gene, such as the coding gene for an exposure that is a protein. For such analyses, the *cis*‐MR design has been suggested to be less prone to confounding from pleiotropy, where variants may be associated with the outcome through their effects on traits other than the exposure (Swerdlow et al., 2016).

Moreover, the potential effect of a drug can be investigated by an MR analysis of a genomic locus encoding protein targets of medicines (Walker et al., 2017). As a result, the *cis*‐MR approach is being increasingly used to provide valuable evidence which can inform designs of expensive clinical trials (Gill et al., 2021). Several examples of *cis*‐MR analyses that have provided insight on the potential efficacy of drug interventions are highlighted in Burgess et al. (2023).

A starting point for any MR analysis is to choose appropriate instruments. Since genome‐wide association studies (GWASs) have been able to identify many strong genetic signals for a wide range of traits, the typical practice in polygenic MR is to select uncorrelated variants with strong measured associations with the exposure.

In contrast, the potential pool of instruments that we can consider for *cis*‐MR is more limited in two aspects. First, the instruments are typically in highly structured correlation, owing to how genetic variants in the same region tend to be inherited together. Second, when the exposure of interest is a gene product, genetic associations are typically measured from much smaller sample sizes than usual GWASs, which would leave *cis*‐MR analyses more vulnerable to problems of weak instrument bias (Andrews et al., 2019). Therefore, for our *cis*‐MR focus, we will need to make use of *many weak* and *correlated* instruments.

One intuitive option is to filter out variants such that only a smaller set of uncorrelated or weakly correlated instruments remain. However, for *cis*‐MR analyses that involve only weak genetic signals, it would seem important to represent the evidence suggested by many variants in the gene region. Another option is to select only those variants with a strong measured association with the exposure. While this might avoid problems relating to weak instruments, estimation could be more vulnerable to a *winner's curse* bias (Goering et al., 2002), resulting in poor inferences if the additional uncertainty from instrument selection is not accounted for (Mounier & Kutalik, 2023).

In this paper, we do not propose selecting specific genetic variants as instruments, but rather genetic factors. We consider a two‐stage approach. In the first stage, we exploit the highly structured nature of genetic correlations in single gene regions to reduce the dimension of genetic variants. Following Bai & Ng (2002)'s approximate factor model, the variation in a large number of genetic variants is assumed to be explained by a finite number of latent factors. The estimated genetic factors are particular linear combinations of genetic variants which aim to capture all systematic variation in the gene region of interest. In the second stage, these estimated genetic factors are used as instrumental variables.

We focus on the problem of providing robust summary‐data inference when the genetic factors are weak instruments. This is a concern not only due to the potentially smaller sample sizes involved in *cis*‐MR analyses, but because the first‐stage dimension reduction of genetic variants is based on their mutual correlation, and not on the strength of their association with the exposure. Thus, there is no guarantee that the estimated genetic factors would be strong instruments.

To provide valid inferences when estimated genetic factors are weak instruments, we consider two different approaches based on conditional testing. The first approach generalizes popular identification‐robust tests (Moreira, 2003; Wang & Kang, 2022) for our setting with estimated genetic factors as instruments. Similar to Bai and Ng (2010)'s analysis under strong instruments, the asymptotic null distributions of the identification‐robust test statistics can be established even when the true genetic factors are not identified.

One drawback with the identification‐robust approaches is that they are unable to provide point estimates of the causal effect. In situations where a few instruments are considerably stronger than others, it is natural to question whether it might be better to discard those instruments which are almost irrelevant. In our case, if some of the estimated genetic factors appear to have very weak associations with the exposure, then we may consider dropping them, and then proceed with usual point estimation strategies. Therefore, in the second approach, we propose a test which appropriately adjusts for first‐stage screening of genetic factors based on their relevance. The test controls the selective type I error: the error rate of a test of the causal effect given the selection of genetic factors as instruments (Fithian et al., 2017; Bi et al., 2020).

Our empirical motivation concerns a potential drug target of growing interest. Cholesteryl ester transfer protein (CETP) inhibitors are a class of drug that increase high‐density lipoprotein cholesterol and decrease low‐density lipoprotein cholesterol (LDL‐C) concentrations. A recent *cis*‐MR analysis by Schmidt et al. (2021) suggests that CETP inhibition may be an effective drug target for reducing coronary heart disease (CHD) risk.

A simulation study based on real *CETP* gene summary data illustrates how both factor‐based conditional testing approaches offer reliable inferences under realistic problems faced in practice: weak instruments, invalid instruments, and mismeasured instrument correlations. Our application complements Schmidt et al. (2021)'s findings by providing robust evidence that the genetically‐predicted LDL‐C lowering effect of CETP inhibitors is associated with a lower risk of CHD.

We use the following notation and abbreviations: $\overset{P}{\to }$ ‘converges in probability to’; $\overset{D}{\to }$ ‘converges in distribution to’; $\overset{a}{∼}$ ‘is asymptotically distributed as’. For any sequences $a_{n}$ and $b_{n}$, if $a_{n}=O\left(b_{n}\right)$, then there exists a positive constant *C* and a positive integer *N* such that for all n≥N, $b_{n}>0$ and $|a_{n}|\leq Cb_{n}$. If $a_{n}=o\left(b_{n}\right)$, then $|a_{n}|/b_{n}\to 0$ as n→∞. Also, if $a_{n}=\Theta \left(b_{n}\right)$, then there exist positive constants *C*_1_ and *C*_2_, $C_{1}\leq C_{2}<\infty$, and a positive integer *N* such that $C_{1}b_{n}\leq a_{n}\leq C_{2}b_{n}$ for all n≥N. Let ${\left(A\right)}_{j}$ denote the *j*th element of any vector **A**, and ${\left(B\right)}_{jk}$ denote the (j,k)th element of any matrix **B**. For any constant *a*, let var(a) denote its population variance. For any vector **A**, let var(A) denote its population variance–covariance matrix. Let ∥.∥ denote the Euclidean norm of a vector. For any positive integer *A*, [A]={1,…,A}. The proofs of the theoretical results are given in Web Appendix C.

## 2 APPROXIMATE FACTOR MODEL AND SUMMARY DATA

Let *X* denote the exposure, *Y* the outcome, and $Z={\left(Z_{1},\text{…},Z_{p}\right)}^{\prime }$ a mean‐centered vector of *p* genetic variants which we assume are valid instrumental variables. For each variant j∈[p], let $\delta_{X_{j}}$ denote the population covariance of variant *j* with the exposure, and $\delta_{Y_{j}}$ denote the population covariance of variant *j* with the outcome. We are interested in estimating the causal effect of the exposure *X* on an outcome *Y*, which is denoted θ_0_ and is described by the linear model

(1) $$\delta_{Y}=\delta_{X}\theta_{0},$$

where $\delta_{X}={\left(\delta_{X_{1}},\text{…},\delta_{X_{p}}\right)}^{\prime }$ and $\delta_{Y}={\left(\delta_{Y_{1}},\text{…},\delta_{Y_{p}}\right)}^{\prime }$. Although this specification does not explicitly allow for variants to have direct effects on the outcome that are not mediated by the exposure, we will later discuss how the approaches we consider are quite robust to this assumption.

### 2.1 Approximate factor model

Our asymptotic framework considers the setting where p→∞, since we aim to incorporate information from many genetic variants. For our *cis*‐MR focus, we would expect a large number of genetic variants to have a block‐like correlation structure (see, e.g., the variant correlations from the *CETP* gene in Figure 3 of Section 5). Therefore, we assume genetic variants in the region of interest follow an approximate factor model structure (Bai & Ng, 2002),

(2) Z=Λf+e,

where $\Lambda ={\left(\lambda_{1},\text{…},\lambda_{p}\right)}^{\prime }$ is an unobserved p×r matrix of factor loadings, **f** is an *r*‐vector of unobserved factors, and $e={\left(e_{1},\text{…},e_{p}\right)}^{\prime }$ is a *p*‐vector of idiosyncratic errors. For each variant *j*, the component ${\lambda_{j}}^{\prime }f$ describes its systematic variation. Although *p* is large, *r* is considered to be finite; the systematic variation of *p* variants can be explained by a much smaller set of *r* latent factors. Thus, instead of using *p* genetic variants as instruments, we will aim to use the information of these *r* latent factors to construct instruments. We note that these latent factors are estimated for a specific gene region, and not for the whole genome; the latter are often used to adjust for the population structure.

Assumption 1 (approximate factor model) (i) The unobserved factors and idiosyncratic errors are identically and independently distributed across individuals; (ii) the factor loadings, factors, and idiosyncratic errors are three mutually independent groups, and the idiosyncratic errors may have some dependence across variants; (iii) the idiosyncratic errors may have limited correlation with the sampling errors of genetic association estimates; (iv) the factors satisfy $E(∥f{∥}^{4})=O\left(1\right)$ and $\Sigma_{F}=E\left({\mathrm{ff}}^{\prime }\right)$ is an r×r positive‐definite matrix; (v) for all variants *j*, $∥\lambda_{j}∥\leq C_{\lambda }$ for some constant $C_{\lambda }>0$, and $p^{-1}\Lambda^{\prime }\Lambda \to \Sigma_{\Lambda }$, where $\Sigma_{\Lambda }$ is a positive‐definite, non‐random matrix, as p→∞.

**FIGURE 1:**
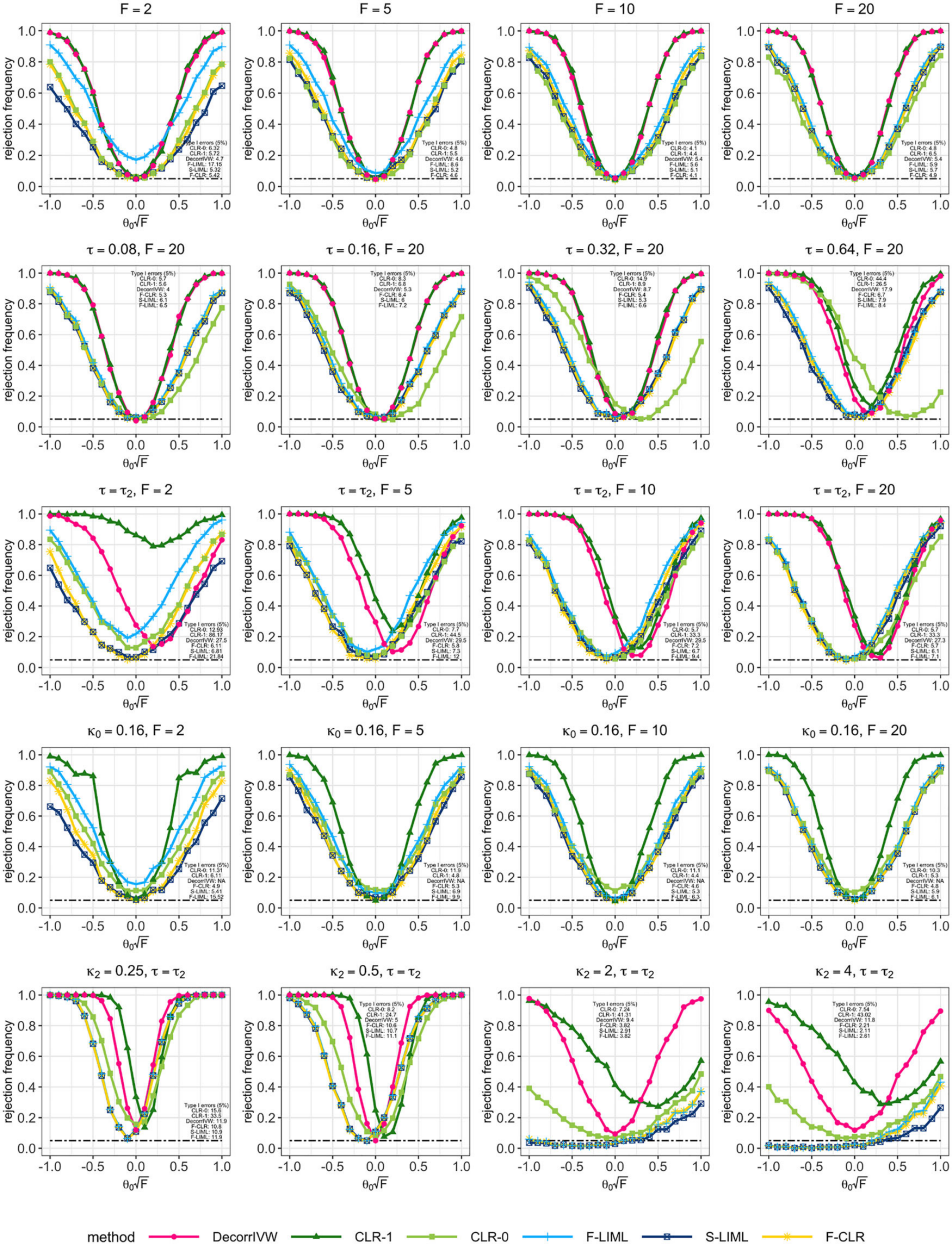
Power results when testing the null hypothesis $H_{0}:\theta_{0}=0$ for a 5% level test. The first row of panels correspond to Model 1 in Section 4.1. The second and third rows of panels correspond to Models 2 and 3 in Section 4.2. The fourth and last rows of panels correspond to Models 4 and 5 in Section 4.3.

**FIGURE 2:**
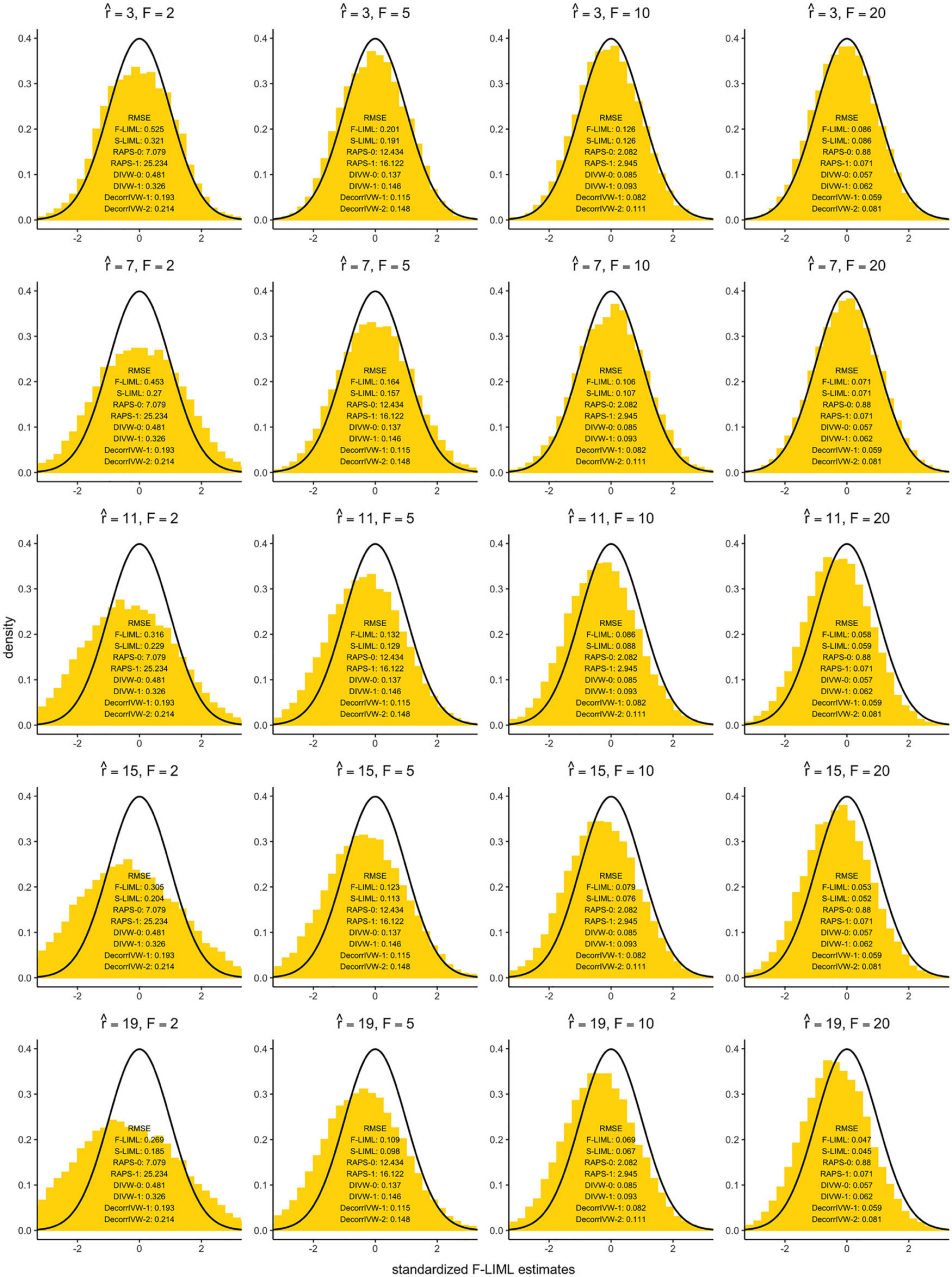
Root‐mean squared error (RMSE) results under locally invalid instruments (Model 3). The histograms correspond to the standardized F‐LIML estimates, and the solid black line is the *N*(0, 1) density curve.

**FIGURE 3:**
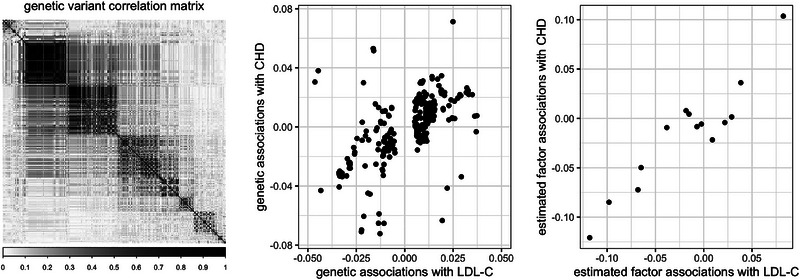
Genetic variant correlations (left), 368 genetic associations with LDL‐C and CHD (center), and 14 estimated factor associations with LDL‐C and CHD (right), in the *CETP* gene region. CHD, coronary heart disease; LDL‐C, low‐density lipoprotein cholesterol.

Assumption 1 implies Assumptions A–F from Bai (2003, pp. 141–144); the assumptions imply that *r* strong factors exist, and that, as p→∞, there is a significant difference between the *r*th and (r+1)th eigenvalues of the variance–covariance matrix of **Z**, var(Z). Throughout our analysis, *r* is considered to be fixed and known. In Section 4.4, we discuss the potential issues with misspecifying the true number of factors.

Compared with classical factor models, the assumptions maintained in an approximate factor model are weak enough to prevent separate identification of factors and factor loadings, however both can be estimated up to an r×r rotation matrix. This should involve no loss of information since in terms of retaining the same explanatory power, we require only that the estimated factors span the same space as the true factors (Bai & Ng, 2002). For details on the dependence permitted between idiosyncratic errors across variants, see Bai and Ng (2010, Assumption A(c), p. 1581). We take the assumptions of the factor model at face value, but in practice we recommend verifying that there is significant structure in the variance–covariance matrix of genetic variants, and that the estimated factors explain a large proportion of genetic variation. Further discussion of Assumption 1(iii) is provided in Web Appendix B.2.

### 2.2 Two‐sample summary data

We work within the popular two‐sample summary data design, where estimated variant–exposure associations ${\hat{\delta }}_{X}={\left({\hat{\delta }}_{X_{1}},\text{…},{\hat{\delta }}_{X_{p}}\right)}^{\prime }$ are obtained from a non‐overlapping, but representative, sample from estimated variant–outcome associations ${\hat{\delta }}_{Y}={\left({\hat{\delta }}_{Y_{1}},\text{…},{\hat{\delta }}_{Y_{p}}\right)}^{\prime }$. Let $n_{X}$ denote the size of the sample used to compute variant–exposure associations, and $n_{Y}$ denote the sample size used to compute variant–outcome associations. We assume that $n:=n_{X}=cn_{Y}$ for some positive constant 0<c<∞. This ensures that for our two‐sample setting, the sampling uncertainty from one association study is not negligible to the other.

For any two variants *j* and *k*, we also assume knowledge of the population genetic correlation $\rho_{jk}$. Genetic correlation estimates can be obtained from popular MR software packages (see, e.g., Hemani et al., 2018). Using a similar approach to Wang & Kang (2022, Theorem 2), we can combine these estimates with GWAS summary data which allow us to construct an estimate of the variance–covariance matrix of genetic variants, $\hat{\text{var}}\left(Z\right)$.

Assumption 2 (summary data on genetic associations) The estimated genetic associations satisfy

$$\left(\begin{array}{c} \Lambda^{\prime }{\hat{\delta }}_{X}\\ \Lambda^{\prime }{\hat{\delta }}_{X} \end{array}\right)∼N\left\{\left(\begin{array}{c} \Lambda^{\prime }\delta_{X}\\ \Lambda^{\prime }\delta_{Y} \end{array}\right),\left(\begin{array}{cc} \Omega_{0}\sigma_{V}^{2}n_{X}^{-1} & 0\\ 0 & \Omega_{0}\sigma_{\varepsilon }^{2}n_{Y}^{-1} \end{array}\right)\right\},$$

where $\sigma_{V}^{2}=var\left(X\right)-{\left(\Lambda^{\prime }\delta_{X}\right)}^{\prime }{\Omega_{0}}^{-1}\left(\Lambda^{\prime }\delta_{X}\right)$, $\sigma_{\varepsilon }^{2}=var\left(Y\right)-{\left(\Lambda^{\prime }\delta_{Y}\right)}^{\prime }{\Omega_{0}}^{-1}\left(\Lambda^{\prime }\delta_{Y}\right)$, and $\Omega_{0}=\Lambda^{\prime }var\left(Z\right)\Lambda$.

Assumption 2 states that the estimated genetic variant–trait covariances are normally distributed around their population counterparts, and that their standard errors are decreasing at the usual parametric rate. This is usually justified by large random sampling in GWASs (Zhao et al., 2020, Assumption 1). Assumption 2 also describes a setting of conditional homoscedastic errors in a linear model, where conditional trait variances given genetic factors are constant. In general, a similar assumption is often maintained for conducting joint analyses from marginal summary genetic data (Yang et al., 2012).

### 2.3 Weak genetic associations

Our asymptotic analysis relies on the assumption that many genetic variants have weak associations with the exposure; see, for example, Zhao et al. (2020).

Assumption 3 (many weak genetic associations) $∥\delta_{X}∥=O\left(1\right)$ as n,p→∞, where $pn^{-1/2}\to \infty$.

Assumption 3 implies that the average explanatory power of any individual variant is decreasing with the total number of variants. Since we do not directly use individual variants as instruments, this does not necessarily imply we face a weak instruments problem. We will take *r* linear combinations of all variants to use as instruments; these linear combinations will correspond to taking the space spanned by the factor loadings Λ, which we can consistently estimate under the rate restriction $pn^{-1/2}\to \infty$, as n,p→∞. As discussed by Bai and Ng (2010), it is possible for these linear combinations to be strong instruments even if the explanatory power of individual variants is limited.

However, for our focus, we deem this to be quite unrealistic: our dimension reduction of genetic variants will be based on their covariance structure, not their association with the exposure. Hence, we could end up using instruments that are able to summarize nearly all genetic variation in a gene region, but they are still weakly associated with the exposure. For this reason, we should focus on inferential methods that are robust to weak instruments.

## 3 CONDITIONAL INFERENCE IN *cis*‐MR WITH GENETIC FACTORS

### 3.1 Estimating the factor loadings

We start by estimating the p×r matrix of factor loadings Λ using the estimated variance–covariance matrix of **Z**, $\hat{\text{var}}\left(Z\right)$. For a given number of factors *r*, let $\bar{\Lambda }$ denote a p×r matrix with its columns given by the eigenvectors corresponding to the largest *r* eigenvalues of $\hat{\text{var}}\left(Z\right)$ multiplied by $\sqrt{p}$. Then, the estimated re‐scaled factor loadings are given by $\hat{\Lambda }=\bar{\Lambda }{\left(p^{-1}{\bar{\Lambda }}^{\prime }\bar{\Lambda }\right)}^{-1/2}$, so that $p^{-1}{\hat{\Lambda }}^{\prime }\hat{\Lambda }=I_{r}$.

For our analysis, the number of factors *r* is assumed to be known. In practice, we may decide on the number of factors by inspecting the scree plot of $\hat{\text{var}}\left(Z\right)$, or by using data‐driven methods as in Onatski (2010).

### 3.2 Point estimation under strong factor associations

Under the linear model (1), we can use our variant–exposure and variant–outcome associations to construct a vector of estimating equations, ${\hat{\delta }}_{Y}-{\hat{\delta }}_{X}\theta =0$. Here, there are *p* estimating equations for 1 unknown θ. Given our estimated factor loadings, we can effectively reduce the degree of over‐identification.

Let $\hat{g}\left(\theta \right)={\hat{\Lambda }}^{\prime }\left({\hat{\delta }}_{Y}-{\hat{\delta }}_{X}\theta \right)$, so that $\hat{g}\left(\theta \right)=0$ provides *r* estimating equations for θ_0_. In other words, $\hat{g}\left(\theta \right)=0$ are the estimating equations implied by using the linear combination of variants ${\hat{\Lambda }}^{\prime }Z$ as instruments. For brevity, we will refer to ${\hat{\Lambda }}^{\prime }Z$ as the *estimated factors*.

First, we construct consistent estimators ${\hat{\sigma }}_{V}^{2}=\hat{\text{var}}\left(X\right)-{\left({\hat{\Lambda }}^{\prime }{\hat{\delta }}_{X}\right)}^{\prime }{\hat{\Omega }}_{0}^{-1}\left({\hat{\Lambda }}^{\prime }{\hat{\delta }}_{X}\right)$ for $\sigma_{V}^{2}$, and ${\hat{\sigma }}_{\varepsilon }^{2}=\hat{\text{var}}\left(Y\right)-{\left({\hat{\Lambda }}^{\prime }{\hat{\delta }}_{Y}\right)}^{\prime }{\hat{\Omega }}_{0}^{-1}\left({\hat{\Lambda }}^{\prime }{\hat{\delta }}_{Y}\right)$ for $\sigma_{\varepsilon }^{2}$, where ${\hat{\Omega }}_{0}={\hat{\Lambda }}^{\prime }\hat{\text{var}}\left(Z\right)\hat{\Lambda }$. Then, we can construct a consistent estimator $\hat{\Omega }\left(\theta_{0}\right)={\hat{\Omega }}_{0}\cdot \left(n_{Y}^{-1}{\hat{\sigma }}_{\varepsilon }^{2}+\theta_{0}^{2}n_{X}^{-1}{\hat{\sigma }}_{V}^{2}\right)$ for $\text{var}(\hat{g}\left(\theta_{0}\right))$. A limited information maximum likelihood (LIML; Anderson and Rubin, 1949) estimator is given by

$${\hat{\theta }}_{F}=\arg\min_{\theta }\hat{g}{\left(\theta \right)}^{\prime }\hat{\Omega }{\left(\theta \right)}^{-1}\hat{g}\left(\theta \right).$$

We call ${\hat{\theta }}_{F}$ the F‐LIML estimator; the LIML estimator which uses the entire vector of estimated factors as instruments.

Theorem 1 Under Assumptions 1–3 and Equations (1) and (2), if $∥\Lambda^{\prime }\delta_{X}∥=\Theta \left(p^{1/2}\right)$, then ${\hat{V}}^{-1/2}\left({\hat{\theta }}_{F}-\theta_{0}\right)\overset{D}{\to }N\left(0,1\right)$ as n,p→∞, where $\hat{V}={\left({\hat{G}}^{\prime }{\hat{\Omega }}^{-1}\hat{G}\right)}^{-1}$, $\hat{G}=-{\hat{\Lambda }}^{\prime }{\hat{\delta }}_{X}$, and $\hat{\Omega }=\hat{\Omega }\left({\hat{\theta }}_{F}\right)$.

The condition $∥\Lambda^{\prime }\delta_{X}∥=\Theta \left(p^{1/2}\right)$ means that the genetic factors are collectively strong instruments, and it requires that many variants have weak effects on the exposure. Theorem 1 can be used directly to construct asymptotic confidence intervals and tests for the causal effect θ_0_.

### 3.3 Identification‐robust tests under weak factor associations

Standard *t*‐tests based on Theorem 1 will not be valid when the estimated factors are weak instruments. This is because under weak instrument asymptotics the distribution of *t*‐tests will depend on a measure of instrument strength (Stock et al., 2002). Instead, identification‐robust tests offer a way to make valid inferences in this setting. The basic idea behind this approach is to construct pivotal test statistics *conditional* on a sufficient statistic for instrument strength. Then, since the conditional distributions of these test statistics do not depend on instrument strength under the null hypothesis, the size of the tests can be controlled under weak instruments.

We can follow previous works by constructing these test statistics as a function of two asymptotically mutually independent statistics $\bar{S}$ and $\bar{T}$, where $\bar{S}$ carries the information of the estimated factors being valid instruments, and where $\bar{T}$ incorporates information on the strength of these instruments. Specifically, under the null hypothesis $H_{0}:\theta =\theta_{0}$, let $\bar{S}=\hat{\Omega }{\left(\theta_{0}\right)}^{-1/2}\hat{g}\left(\theta_{0}\right)$, and $\bar{T}={\left\{{\hat{\Delta }}_{\mathrm{GG}}-{\hat{\Delta }}_{G}\hat{\Omega }{\left(\theta_{0}\right)}^{-1}{\hat{\Delta }}_{G}\right\}}^{-1/2}\left\{\hat{G}-{\hat{\Delta }}_{G}\hat{\Omega }{\left(\theta_{0}\right)}^{-1}\hat{g}\left(\theta_{0}\right)\right\}$, where ${\hat{\Delta }}_{\mathrm{GG}}={\hat{\Omega }}_{0}n_{X}^{-1}{\hat{\sigma }}_{V}^{2}$, ${\hat{\Delta }}_{G}={\hat{\Delta }}_{\mathrm{GG}}\theta_{0}$, and where $\hat{\Omega }\left(\theta_{0}\right)$, ${\hat{\Omega }}_{0}$, and ${\hat{\sigma }}_{V}^{2}$ are defined in Section 3.2.

Using $\bar{S}$ and $\bar{T}$, we can construct three commonly‐used identification‐robust test statistics which will be asymptotically pivotal conditional on $Z_{T}∼N\left\{{\left(\Delta_{\mathrm{GG}}-\Delta_{G}\Omega^{-1}\Delta_{G}\right)}^{-1/2}G,I_{r}\right\}$, where $\Delta_{\mathrm{GG}}=H^{-1}\Omega_{0}H^{-1\prime }n_{X}^{-1}\sigma_{V}^{2}$, $\Delta_{G}=\Delta_{\mathrm{GG}}\theta_{0}$, $\Omega =H^{-1}\Omega_{0}H^{-1\prime }\cdot \left(n_{Y}^{-1}\sigma_{\varepsilon }^{2}+\theta_{0}^{2}n_{X}^{-1}\sigma_{V}^{2}\right)$, $G=-H^{-1}\Lambda^{\prime }\delta_{X}$, and **H** is a rotation matrix. Let ${\bar{Q}}_{S}={\bar{S}}^{\prime }\bar{S}$, ${\bar{Q}}_{ST}={\bar{S}}^{\prime }\bar{T}$, and ${\bar{Q}}_{T}={\bar{T}}^{\prime }\bar{T}$. Then, the Anderson and Rubin (1949) statistic with estimated factors, denoted F‐AR, is given by $\text{F-AR}={\bar{Q}}_{S}$, Kleibergen (2005)'s Lagrange multiplier statistic with estimated factors, denoted F‐LM, is given by $\text{F-LM}={\bar{Q}}_{ST}^{2}/{\bar{Q}}_{T}$, and Moreira (2003)'s conditional likelihood ratio statistic with estimated factors, denoted F‐CLR, is given by $\text{F-CLR}=[{\bar{Q}}_{S}-{\bar{Q}}_{T}+{\left\{{\left({\bar{Q}}_{S}+{\bar{Q}}_{T}\right)}^{2}-4\left({\bar{Q}}_{S}{\bar{Q}}_{T}-{\bar{Q}}_{ST}^{2}\right)\right\}}^{1/2}]/2$.

Theorem 2 Suppose that $\Lambda^{\prime }\delta_{X}=\Theta \left(n^{-1/2}p^{1/2}\right)$ and $\delta_{X_{k}}=O\left(n^{-a}p^{-1/2}\right)$, k∈[p], for some a>0. Under Assumptions 1–3, Equations (1) and (2), and $H_{0}:\theta =\theta_{0}$, conditional on $Z_{T}$, (i) $\text{F-AR}\overset{D}{\to }\chi_{r}^{2}$; (ii) $\text{F-LM}\overset{D}{\to }\chi_{1}^{2}$; and (iii) $\text{F-CLR}\overset{D}{\to }\left[\chi_{1}^{2}+\chi_{r-1}^{2}-Z_{T}^{\prime }Z_{T}+{\left\{{\left(\chi_{1}^{2}+\chi_{r-1}^{2}-Z_{T}^{\prime }Z_{T}\right)}^{2}+4\chi_{1}^{2}Z_{T}^{\prime }Z_{T}\right\}}^{1/2}\right]/2$ as n,p→∞, where $\chi_{1}^{2}$ and $\chi_{r-1}^{2}$ denote independent chi‐square random variables.

The rate restriction $\Lambda^{\prime }\delta_{X}=\Theta \left(n^{-1/2}p^{1/2}\right)$ describes the setting where all genetic factors are weak instruments. The condition allows for all variant associations with the exposure to be collectively weak $∥\delta_{X}∥=\Theta \left(n^{-1/2}\right)$ as long as they are not too uneven. Since the F‐AR statistic is not a function of $\bar{T}$, it does not incorporate the identifying power of instruments. As a result, when the model is over‐identified (r>1), the F‐AR test may have relatively poor power properties compared with the F‐LM and F‐CLR tests (Andrews et al., 2019). Of the three methods, CLR‐based tests are widely regarded as the most powerful, due to simulation evidence and favorable theoretical properties (Andrews et al., 2006). To implement the CLR test, we use the algorithm for computing its asymptotic conditional *p*‐value derived by Andrews et al. (2007).

### 3.4 Conditional tests that adjust for factor selection

While identification‐robust approaches are designed to control type I error rates for any level of instrument strength, they do not provide point estimates. In a sparse effects setting where a few estimated factors are strong instruments and most other estimated factors are very weak instruments, it would be tempting to proceed with F‐LIML point estimation after removing very weak instruments. The approaches described in Sections 3.2 and 3.3 use the entire *r*‐vector of estimated factors as instruments. In contrast, here we wish to filter out certain elements if they are demonstrably weak instruments.

To this end, we construct pre‐tests to identify a subset of estimated factors that pass a threshold of relevance; only this subset is then used as instruments. By Bai (2003), $\hat{\Lambda }$ estimates $\Lambda H^{\prime -1}$ where **H** is a rotation matrix. Hence, the estimated factor associations ${\hat{\Lambda }}^{\prime }{\hat{\delta }}_{X}$ actually estimate $H^{-1}\Lambda^{\prime }\delta_{X}$, and not $\Lambda^{\prime }\delta_{X}$ as we may intuitively expect. Therefore, for each j∈[r], we will test the null hypothesis $H_{0j}:{\left(G\right)}_{j}=0$ against the alternative $H_{1j}:{\left(G\right)}_{j}\neq 0$, where $G=-H^{-1}\Lambda^{\prime }\delta_{X}$.

Simple *t*‐tests are used to screen for relevant estimated factors, using the asymptotic approximation ${\left({\hat{\Delta }}_{\mathrm{GG}}\right)}_{jj}^{-1/2}{\left(\hat{G}-G\right)}_{j}\overset{a}{∼}N\left(0,1\right)$, j∈[p]. In particular, to conduct a two‐sided asymptotic υ‐level test for the significance of each estimated factor j∈[r], we compare the test statistic $|{\hat{T}}_{j}|$, where ${\hat{T}}_{j}={\left({\hat{\Delta }}_{\mathrm{GG}}\right)}_{jj}^{-1/2}{\left(\hat{G}\right)}_{j}$, against the critical value $c_{\upsilon }:=\Phi \left(1-\upsilon /2\right)$, where Φ(.) is the standard normal cumulative distribution function. For each j∈[r], if $|{\hat{T}}_{j}|>c_{\upsilon }$, then we have evidence to reject $H_{0j}$, and thus we include the estimated factor *j* as an instrument. Any estimated factors such that $|{\hat{T}}_{j}|\leq c_{\upsilon }$ are deemed to be weak instruments, and are thus discarded.

Let S denote the selection event according to these pre‐tests. For example, if r=3 and only the first and third estimated factors pass the pre‐test of relevance, then $S=\{|{\hat{T}}_{1}|>c_{\upsilon },|{\hat{T}}_{2}|\leq c_{\upsilon },|{\hat{T}}_{3}|>c_{\upsilon }\}$. Only using the subset of estimated factors that have passed the pre‐test of relevance, we will test the null hypothesis $H_{0}:\theta =\theta_{0}$ against the general alternative $H_{1}:\theta \neq \theta_{0}$. To appropriately account for pre‐testing of relevant estimated factors, we seek to construct a conditional test which controls the selective type I error (Fithian et al., 2017). That is, for an α‐level test, $P(\text{reject}\;H_{0}|S)\leq \alpha$ under $H_{0}$; that is, we control the error rate of the test at α*given* the selection event S.

Suppose $r^{★}$ is the number of selected estimated factors, and let R denote the indices of the selected set of estimated factors, so that the selection event is $S=\left\{\right|{\hat{T}}_{j}|>c_{\upsilon },j\in R\}\cap \{|{\hat{T}}_{k}|\leq c_{\upsilon },k\in \left[r\right]\setminus R\}$. We also let $\Gamma_{S}$ denote an $r\times r^{★}$ selection matrix which is constructed such that $\hat{\Lambda }\Gamma_{S}$ is the $p\times r^{★}$ matrix, where its $r^{★}$ columns are the columns of $\hat{\Lambda }$ that correspond to the selected factors R. We call the resulting LIML estimator which uses only the selected factors ‘Selected LIML’ (S‐LIML), which is denoted as ${\hat{\theta }}_{S}$.

We are often interested in testing the null hypothesis $H_{0}:\theta =\theta_{0}$ for non‐zero values $\theta_{0}\neq 0$; for example, this is useful for obtaining confidence intervals by test inversion. In this case, we would need to consider how the distribution of ${\hat{\theta }}_{S}$ is impacted by the uncertainty of the pre‐test results S (see Web Appendix D.1 for further discussion). Let $\Omega_{S}=\Gamma_{S}^{\prime }\Omega \Gamma_{S}$, $G_{S}=\Gamma_{S}^{\prime }G$, and $V_{S}={\left(G_{S}^{\prime }\Omega_{S}^{-1}G_{S}\right)}^{-1}$. We base our inferences on the following joint normality approximation which partially describes the dependency of ${\hat{\theta }}_{S}$ on the vector of pre‐test statistics $\hat{T}={\left({\hat{T}}_{1},\text{…},{\hat{T}}_{r}\right)}^{\prime }$,

(3) $$\left(\begin{array}{c} {\hat{\theta }}_{S}\\ \hat{T} \end{array}\right)\overset{\text{approx.}}{∼}N\left\{\left(\begin{array}{c} \theta_{0}\\ D^{-1/2}G \end{array}\right),\left(\begin{array}{cc} V_{S} & {C_{G}}^{\prime }\\ C_{G} & V_{G} \end{array}\right)\right\},$$

where $C_{G}=-D^{-1/2}\Delta_{\mathrm{GG}}\Gamma_{S}\Omega_{S}^{-1}G_{S}V_{S}\theta_{0}$, $V_{G}=D^{-1/2}\Delta_{\mathrm{GG}}D^{-1/2}$, and **D** is the diagonal matrix with its (j,j)th element given by ${\left(\Delta_{\mathrm{GG}}\right)}_{jj}$. We take this approximation as given, but it is not exact because we do not account for the estimation error term $\hat{T}-D^{-1/2}\bar{G}$, for $\bar{G}=-H^{-1}\Lambda^{\prime }{\hat{\delta }}_{X}$, which may not be negligible under the setting where the estimated factors are strong instruments. However, in our simulation experiments, this approximation performs reasonably well under weak and strong instrument settings. Equation (3) suggests that the conditional distribution of ${\hat{\theta }}_{S}$ given S depends on an *r*‐dimensional nuisance parameter $D^{-1/2}G$. Thus, along with the selection event S, we will condition on a sufficient statistic for the nuisance parameter, which will cause it to drop from the conditional distribution of ${\hat{\theta }}_{S}$ (see, e.g., Sampson & Sill, 2005). According to (3), a sufficient statistic for $D^{-1/2}G$ is given by $U=\hat{T}-C_{G}V_{S}^{-1}{\hat{\theta }}_{S}$.

Theorem 3 Under Equation (3) and $H_{0}:\theta =\theta_{0}$, the conditional distribution of ${\hat{\theta }}_{S}$ given U=u and S is approximately

(4) $$\begin{array}{ccc} & & P({\hat{\theta }}_{S}\leq w|S,U=u)\\ & & =\frac{P\left(\left\{\theta_{0}+V_{S}^{1/2}K\leq w\right\}\cap_{j\in R}\left\{\right|{\left(\bar{u}\right)}_{j}|>c_{\upsilon }\}\cap_{k\in [r]\setminus R}\left\{\right|{\left(\bar{u}\right)}_{k}\left|\leq c_{\upsilon }\right\}\right)}{P\left(\cap_{j\in R}\left\{\right|{\left(\bar{u}\right)}_{j}|>c_{\upsilon }\}\cap_{k\in [r]\setminus R}\left\{\right|{\left(\bar{u}\right)}_{k}\left|\leq c_{\upsilon }\right\}\right)}, \end{array}$$

where $\bar{u}=u+C_{G}V_{S}^{-1/2}K$, and K∼N(0,1).

Intuitively, this conditional distribution of ${\hat{\theta }}_{S}$ reveals what the likely values of ${\hat{\theta }}_{S}$ *should* be under $H_{0}:\theta =\theta_{0}$, given the results of the pre‐tests S and observed value U=u. If the S‐LIML estimate ${\hat{\theta }}_{S}$ does not lie in a suitable likely region, then we interpret this as evidence against $H_{0}$.

We can construct estimators ${\hat{C}}_{G}=-{\hat{D}}^{-1/2}{\hat{\Delta }}_{\mathrm{GG}}\Gamma_{S}{\hat{\Omega }}_{S}^{-1}{\hat{G}}_{S}{\hat{V}}_{S}{\hat{\theta }}_{S}$ of **C_G_**, ${\hat{V}}_{S}={\left({\hat{G}}_{S}^{\prime }{\hat{\Omega }}_{S}^{-1}{\hat{G}}_{S}\right)}^{-1}$ of $V_{S}$, where ${\hat{G}}_{S}=\Gamma_{S}^{\prime }\hat{G}$, ${\hat{\Omega }}_{S}=\Gamma_{S}^{\prime }\hat{\Omega }\left({\hat{\theta }}_{S}\right)\Gamma_{S}$, and $\hat{D}$ is the diagonal matrix with its (j,j)th element given by ${\left({\hat{\Delta }}_{\mathrm{GG}}\right)}_{jj}$. By conditioning on $u=\hat{T}-{\hat{C}}_{G}{\hat{V}}_{S}^{-1}{\hat{\theta }}_{S}$, we can conduct an approximate α‐level test for $H_{0}:\theta =\theta_{0}$ by using the sample analog of the right‐hand side of Equation (4), taking repeated draws of K∼N(0,1), and computing α/2 and (1−α/2)‐level quantiles of the approximated $P({\hat{\theta }}_{S}\leq w|S,U=u)$ distribution under $H_{0}$. If the S‐LIML estimate ${\hat{\theta }}_{S}$ does not lie within those quantiles, then we reject the null hypothesis $H_{0}$.

## 4 SIMULATION STUDY

This section presents the performance of the identification‐robust and conditional test statistics in a simulation study based on real genetic data. The simulation design aims to explore the robustness of our empirical results in Section 5, where we investigate CETP inhibitors as a potential drug target for CHD. CETP are a class of drug which increase high‐density lipoprotein cholesterol and decrease LDL‐C. Our exposure *X* is LDL‐C, our outcome *Y* is CHD, and to construct instruments we used p=180 genetic variants from a neighborhood of the *CETP* gene which are associated with LDL‐C at *p*‐value less than $5\times 10^{-2}$.

The true factor loadings were set as the factor loadings corresponding to the measured variance–covariance matrix of genetic variants in the CETP region, and the true variant–trait associations $\delta_{X}$ and $\delta_{Y}$ were taken as the measured variant associations with LDL‐C and CHD. We generated ${\hat{\delta }}_{X}$ and ${\hat{\delta }}_{Y}$ according to Assumption 2 and Equation (1). The unconditional trait variances were set equal to 1, and the sample sizes for both association studies were set equal to *n*, where *n* was chosen to vary the instrument strength of genetic factors according to a particular value of the *F*‐statistic. Unless otherwise stated, the *F*‐statistic was set equal to 20, and the true number of factors was set to r=11.

The variant correlation matrix ρ was held fixed and equal to the measured correlation matrix from the *CETP* gene. For our proposed methods, we generated sampling errors in estimation of the variance–covariance matrix var(Z). In particular, for each variant *j*, we only observed $\hat{\text{var}}\left(Z_{j}\right)$ which was generated as a truncated normal with mean $\text{var}(Z_{j})$, variance $n^{-1}$, bounded by the minimum and maximum measured $\text{var}(Z_{k})$ over all variants k∈[p].

Our proposed methods are based on dimension reduction of all variants rather than selecting specific variants. Instead of using estimated factors as instruments, we might wonder if it is better to omit highly correlated variants and use existing approaches which account for a smaller number of moderately or nearly uncorrelated correlated variants as instruments. Thus, for comparison we note the results of Wang & Kang (2022)'s CLR test, where variants are filtered out if they are correlated with an already included variant at some pre‐specified threshold. In MR terminology, this is called *pruning*. Another way to incorporate moderately correlated variants is to de‐correlate summary data associations using knowledge of the variant–correlation matrix ρ, and then apply simpler MR estimation strategies such as inverse variance weighted (IVW; Burgess et al., 2013). DecorrIVW notes the results of a de‐correlated IVW method using a pruning threshold of $R^{2}\leq 0.1$.

Finally, we also consider approaches which assume uncorrelated variants, but are robust to many weak instruments and outlier effects, such as robust adjusted profile Score (RAPS; Zhao et al., 2020), De‐biased IVW (DIVW; Ye et al., 2021), and a radial IVW regression approach with second‐order weights (RAD, Bowden et al., 2019). For indicating the level of pruning used, the tests CLR‐0, CLR‐1, CLR‐2, and CLR‐4 will denote the CLR test pruned to near‐independence after selecting the strongest associated variant with LDL‐C ($R^{2}\leq 0.01$), $R^{2}\leq 0.1$, $R^{2}\leq 0.2$, and $R^{2}\leq 0.4$, respectively. Likewise, RAPS‐0, RAPS‐1, and other tests are defined analogously.

Our simulation study focuses on studying the performance of tests under three practical problems of interest in *cis*‐MR analyses: weak instruments, invalid instruments, and mismeasured instrument correlations. The full results from all methods are given in Web Appendix D.

### 4.1 Weak instruments

Since proteins are the drug target of most medicines, *cis*‐MR analyses often use proteins as the exposure of interest. Genetic associations with protein or gene expression are typically measured with smaller sample sizes than usual GWASs. In practice, this can result in a weak instruments problem. In Model 1, we explore the performance of tests under correct model specification and varying instrument strength, as measured by the *F*‐statistic of the association between the true genetic factors $\Lambda^{\prime }Z$ and *X*.

The first row in Figure 1 shows that when all variants are valid instruments, using moderately correlated individual variants as instruments performs well; the CLR‐1 and DecorrIVW tests are considerably more powerful than the factor‐based tests even under a pruning threshold of $R^{2}\leq 0.1$. Using estimated factors as instruments provide reliable inference for both F‐CLR and S‐LIML approaches, with both tests offering slightly more power than CLR‐0 under strong instruments. The S‐LIML test screened the estimated factors for relevance using υ=0.01 level pre‐tests for F≥10, υ=0.05 for F=5, and υ=0.1 for F=2. With these thresholds, the median number of factors retained were $r^{★}=4$ under F=2, and $r^{★}=7$ under F=20. The first row in Figure 1 further suggests that under weak factor instruments (F<10), F‐LIML which uses all 11 estimated factors does not control type I error rates. Although LIML estimators may provide consistent estimation under many weak instruments (Chao & Swanson, 2005), for inference, the conventional standard errors are too small (Newey & Windmeijer, 2009).

### 4.2 Invalid instruments

Although the linear model (1) is commonly used in practice, we may be concerned that proportionality of genetic associations $\delta_{Y_{j}}=\theta_{0}\delta_{X_{j}}$ may not hold exactly over all variants j∈[p]. Fortunately, the factor model approach may provide some robustness to the inclusion of invalid instruments. For example, under Bai and Ng (2010)'s analysis, a finite number of variants would be permitted to have direct effects on the outcome as long as the total number of variants *p* grows sufficiently faster than *n*.

Here, we study finite‐sample behavior under local model misspecification $\delta_{Y}=\delta_{X}\theta_{0}+n^{-1/2}\tau$, where the direct effects τ are fixed. Under this setting, the squared bias and variance terms of the F‐LIML estimator that comprise its mean squared error are of the same order of magnitude asymptotically. In Model 2, these direct effects are equal for all variants, and the results are displayed in the second row of Figure 1. For τ≤0.16, the CLR‐1 and DecorrIVW tests which use moderately correlated individual variants as instruments offer competitive size properties along with the factor‐based approaches. However, for more severe problems of directional pleiotropy (τ>0.32), F‐CLR and S‐LIML are better able to control type I error rates.

In Model 3, the direct effects are generated as $\tau =\tau_{2}$, where each element of $\tau_{2}$ is drawn from the uniform distribution U(−1,1), and we vary instrument strength with the *F*‐statistic. This is similar to an assumption of balanced pleiotropy often maintained in polygenic MR (Hemani et al., 2018). When the direct effects τ are random around zero, their impact is to inflate the variance of the resulting estimate. In contrast, here we set the direct effects τ to be fixed, so that they directly impact the bias of the resulting estimates, with no de‐biasing adjustment possible without imposing further restrictions. The results from Model 3 are displayed in the third row of Figure 1, and they further highlight the robust performance of F‐CLR and S‐LIML under invalid instruments, with relatively small‐size distortions regardless of instrument strength.

### 4.3 Mismeasured variant correlations

In Models 4 and 5, our simulation designs investigate robustness to a very common problem in *cis*‐MR analyses. It is often the case that the variant correlation matrix ρ is not provided alongside summary genetic association data. In such situations, if researchers want to make use of correlated variants, they would need to obtain estimates of the variant correlation matrix from a reference sample containing a different set of subjects.

Discrepancies between the variant correlation matrix from the reference sample $\bar{\rho }$, and the true variant correlation matrix for the two‐sample summary data ρ may arise due to at least two reasons. First, the size of the reference sample may be significantly lower than the sample size of GWASs, thus allowing more room for sampling errors. Second, while all samples are assumed to be drawn from the same population, in practice the two correlation estimates may be based on heterogeneous samples.

To study the problem of mismeasured variant correlations, in Model 4, for any two variants j,k, we assume the variant correlation estimate available to the researcher satisfies ${\bar{\rho }}_{jk}=\rho_{jk}-\kappa_{0}$, where $\rho_{jk}$ are the true variant correlations used to construct the two‐sample summary associations, and κ_0_ is a fixed constant. Here, we set where $\kappa_{0}=0.16$ since the minimum observed value in the variant correlation matrix was −0.839, so that ${\bar{\rho }}_{jk}=\rho_{jk}-0.16$ still returns feasible correlation values. The fourth row of Figure 1 displays the results for Model 4, where we note that CLR‐0 which erroneously assumes variants are nearly uncorrelated has inflated type I error rates. It was not possible to note the performance of DecorrIVW due to numerical instabilities, which suggests that an approach based on de‐correlating genetic associations may be more sensitive to mismeasured variant correlations compared with methods that are designed to account for the variant correlation structure.

The design in Model 5 aims to shed light on the potential problems with misspecifying the correlation structure of ρ, which is of practical concern if we suspect that genetic correlations are measured from a population which is not representative of participants from which genetic–trait associations are obtained. We assume that the measured variant correlation matrix available to the researcher is ${\bar{\rho }}_{jk}=sgn\left(\rho_{jk}\right)\cdot {\left|\rho_{jk}\right|}^{\kappa_{2}}$, for all j,k∈[p], and $\kappa_{2}>0$. If $\kappa_{2}>1$, moderate variant correlations are pushed toward zero, which may result in a fewer number of factors explaining a large proportion of genetic variation. Under Model 5, we selected the number of factors based on identifying an eigenvalue gap in the scree plot. Instead of r=11, we used five estimated factors under $\kappa_{2}=2$, and four estimated factors under for $\kappa_{2}=4$. The results are displayed in final row of Figure 1. Under $\kappa_{2}>1$, F‐CLR, S‐LIML, and F‐LIML have very poor power properties, partially owing to an under‐selection of estimated factors. For Model 5, CLR‐0 appears to achieve a good balance between type I error control and power. Intuitively, we would prefer filtering to near‐independence rather than use correlated variants based on a largely misspecified correlation structure.

### 4.4 Misspecifying the number of factors

In this section, we discuss the potential problems with misspecifying the true number of factors *r*, which are assumed to be known throughout our analysis. Here, we study the same local misspecification design as Model 3 discussed in Section 4.2, and vary the assumed number of factors $\hat{r}$ away from r=11. The histograms from Figure 2 show the standardized F‐LIML estimates ${\hat{V}}^{-1/2}\left({\hat{\theta }}_{F}-\theta_{0}\right)$ which should be asymptotically distributed as *N*(0, 1) according to Theorem 1. The results show that under‐selecting the number of factors ($\hat{r}<11$) still returns median‐unbiased estimates, whereas using a higher number of factors appears to introduce some bias. For higher values of $\hat{r}$, we run the risk of additional factors prioritizing the information of variants which are in less structured correlation. Under Model 3, many such variants could have strong direct effects on the outcome which may cause estimates to become biased.

Interestingly, over‐selecting the number of factors still results in improved estimation in terms of root‐mean squared error (RMSE); allowing a small amount of bias is a price worth paying for significant gains in variance reduction. Compared with $\hat{r}=3$, the RMSE of F‐LIML is halved when selecting $\hat{r}=19$ factors under very weak instruments (F=2). Especially under weak instrument settings (F<10), S‐LIML outperforms F‐LIML in terms of RMSE, which further underscores the importance of filtering out weak estimated factors. When $\hat{r}\geq 11$, S‐LIML appears to be the best choice for point estimation compared to alternative robust methods. More generally, the DecorrIVW and DIVW methods under strong pruning $\left(R^{2}\leq 0.1\right)$ also provide precise estimates. We also find that over‐estimating the true number of factors may lead to only modest increases in type I error rates for the F‐CLR and S‐LIML methods under Model 5 (see Figure S.9 in Web Appendix D). In practice, we suggest that it may be preferable to lean toward over‐selecting the number of factors rather than under‐selecting based on MSE considerations.

## 5 EMPIRICAL APPLICATION: CHOLESTERYL ESTER TRANSFER PROTEIN INHIBITION AND CORONARY HEART DISEASE

CETP inhibitors are a class of drug which increase high‐density lipoprotein cholesterol and decrease LDL‐C concentrations. At least three CETP inhibitors have failed to provide sufficient evidence of a protective effect on CHD in clinical trials, before the successful trial of Anacetrapib showed marginal benefits alongside statin therapy (Bowman et al., 2017). However, with further trials currently ongoing, *cis*‐MR analyses can offer important supporting evidence to complement experimental results. For example, in recent work, Schmidt et al. (2021)'s *cis*‐MR analysis suggests that CETP inhibition may be an effective drug target for CHD prevention.

From a statistical perspective, we may have a few concerns regarding the criteria used by Schmidt et al. (2021) to select instruments. First, to guard against weak instrument bias, they select variants based on an in‐sample measure of instrument strength (*F*‐statistic > 15), which could potentially leave the analysis vulnerable to a winner's curse bias (Mounier & Kutalik, 2023). Second, to guard against heterogeneity of genetic associations, they use a measure of instrument validity to remove outliers (Cochran's *Q* statistic; see, e.g., Bowden et al., 2019), which can result in size‐distorted tests (Guggenberger & Kumar, 2012). Finally, for those correlated variants with strong measured associations with the outcome, they allow variants up to a pruning threshold of $R^{2}\leq 0.4$; our simulation results in Web Appendix D show that inference can be sensitive to the choice of a pruning threshold.

Here, we apply conditional inference techniques to investigate the genetically‐predicted LDL‐C lowering effect of CETP inhibition on the risk of CHD. Genetic associations with LDL‐C were taken from a GWAS of 361,194 individuals of white‐British genetic ancestry in the UK Biobank and were in standard deviation units (Sudlow et al., 2015). Genetic associations with CHD, measured in log odds ratio units, were taken from a meta‐GWAS of 48 studies with a total of 60,801 cases and 123,504 controls from a majority European population, conducted by the CARDIoGRAMplusC4D consortium (Nikpay et al., 2015). Genetic variant correlations were obtained from a reference panel of European individuals (1000 Genomes Project, Auton et al., 2015) using the twosampleMR R package (Hemani et al., 2018).

Since our method assumes a linear model, we transformed the measured variant–CHD associations that were obtained under a univariable logit regression to approximate estimated coefficients from a univariable linear regression. Further details on this transformation are provided in Web Appendix A.

A total of 368 genetic variants were drawn from the *CETP* region, with variant positions within ±100 kb from the *CETP* gene position indicated on GeneCards (Stelzer et al., 2016). The variant correlation matrix was highly structured, with r=14 factors explaining nearly 99% of the total variation of the 368 genetic variants. Noting the gap between the 14th and 15th eigenvalue, we selected r=14 estimated factors as instruments for the F‐AR, F‐LM, F‐CLR, and S‐LIML methods.

Only 6 of the 14 estimated factors were retained by the S‐LIML method after pre‐testing for relevant factors at the υ=0.01 level. Table 1 shows that the S‐LIML method gives a point estimate of ${\hat{\theta }}_{S}=0.133$ for a unit increase in the risk of CHD associated with a 1 standard deviation change in LDL‐C, with a corresponding 95% confidence interval [0.051,0.215]. The results were reasonably robust to the choice of pre‐testing threshold υ used to select relevant estimated factors, however the S‐LIML estimate becomes less precise for the stricter threshold υ=0.001 which may be due to the exclusion of a relevant factor.

**TABLE 1:** Est. gives the point estimate of the method if applicable.

	${\hat{\theta }}_{F}$	${\hat{\theta }}_{S}\left(0.1^{1}\right)$	${\hat{\theta }}_{S}\left(0.1^{2}\right)$	${\hat{\theta }}_{S}\left(0.1^{3}\right)$	F‐AR	F‐LM	F‐CLR
Est.	0.131	0.132	0.133	0.129	‐	‐	‐
CI‐L	0.052	0.052	0.051	0.045	‐0.050	0.052	0.051
CI‐U	0.210	0.211	0.215	0.220	0.334	0.213	0.215
*Q*‐stat.	0.997	0.997	0.964	0.945	0.998*	0.999*	0.999*

*Note*: CI‐L and CI‐U are the lower and upper estimated 95% confidence intervals. The brackets after S‐LIML indicate the threshold level υ taken. Q-stat gives the *p*‐value associated with testing the null of no heterogeneity in instrument–LDL‐C and instrument–CHD associations using the Sargan–Hansen test; see the discussion in Section 5.

In Table 1, the confidence intervals for S‐LIML and identification‐robust methods are obtained by test inversion. The 95% asymptotic confidence intervals for the F‐CLR and F‐LM tests are similar to the S‐LIML intervals, while the F‐AR approach is much less precise and is unable to reject the null hypothesis of no causal association (F‐AR *p*‐value: 0.455).

The results of alternative summary data methods are presented in Table 2. We find that DecorrIVW and CLR with correlated variants are quite sensitive to the pruning threshold chosen, with the 95% asymptotic confidence interval of DecorrIVW‐1 not overlapping with the DecorrIVW‐2 interval.

Our simulation study illustrated how our factor‐based approaches were relatively robust to biases from direct variant effects on the outcome. The heterogeneity plots in Figure 3 can provide insight on the coherency of evidence across multiple instruments. A more formal method to test for excessive heterogeneity uses the Sargan–Hansen (1982) test. Table 1 shows that the F‐LIML and S‐LIML approaches provide strong evidence of no heterogeneity when using estimated factors as instruments. Since identification‐robust methods do not provide point estimates, for the starred entries in the last rows of Tables 1 and 2, we evaluated the Sargan–Hansen test statistic (1982) at the mid‐point of the relevant confidence interval. There was no ‘degrees of freedom’ correction for this substitution which should lead to more conservative *p*‐values (i.e., we are less likely to reject the null of no heterogeneity). Despite this, more liberal pruning‐based approaches show evidence of greater heterogeneity when considering individual variants as instruments.

**TABLE 2:** Est. gives the point estimate of the method if applicable.

	CLR‐0	CLR‐1	CLR‐2	CLR‐4	RAPS‐0	DIVW‐0	DecorrIVW‐1	DecorrIVW‐2
Est.	–	–	–	–	0.122	0.120	0.068	0.117
CI‐L	0.056	0.021	0.088	0.107	0.081	0.080	0.043	0.101
CI‐U	0.218	0.120	0.153	0.147	0.163	0.160	0.092	0.133
*Q*‐stat.	0.282*	0.610*	0.391*	0.012*	0.252	0.252	0.413	0.000

*Note*: CI‐L and CI‐U are the lower and upper estimated 95% confidence intervals. Q-stat gives the *p*‐value associated with testing the null of no heterogeneity in instrument–LDL‐C and instrument–CHD associations using the Sargan–Hansen test; see the discussion in Section 5.

Overall, our findings provide robust evidence that genetically‐predicted lower LDL‐C levels using variants in the *CETP* gene region are associated with a lower risk of CHD.

## 6 CONCLUSION

There is an increasing focus on using *cis*‐MR analyses to guide drug development; genetic evidence may be crucial to support novel targets, precision medicine subgroups, and the design of expensive clinical trials (Gill et al., 2021). The use of a few uncorrelated variants as instruments may lead to inferences which are vulnerable to direct variant effects on the outcome. On the other hand, using correlated variants as instruments may result in unstable inferences which are particularly sensitive to common problems of misspecified variant correlations. We believe that our factor‐based approach provides a robust and practical way to assess the general weight of evidence for a causal association from the gene region of interest. Given its reliable performance in simulation, we recommend the use of the F‐CLR test alongside existing robust methods in *cis*‐MR investigations, especially in settings where there are multiple genetic signals for the exposure in the gene region.

A limitation of our approach is that we require the knowledge of a large genetic correlation matrix of the gene region, and systematic misspecification of these correlations (e.g., due to structural differences in the two sampled populations) may lead to biased inferences. At the same time, in settings where all variants are valid instruments, methods which use individual variants as instruments, rather than genetic factors, tend to provide a more powerful analysis, especially in cases where there are only a few strong genetic signals for the exposure. Furthermore, when the quality of available genetic correlation estimates is in doubt or there is only one strong genetic signal, it may be sensible to compare results from methods using correlated variants to those of the Wald ratio estimator based on the variant most strongly associated with the exposure. Finally, our approach assumes that the true number of factors is known. We leave for future the work the problem of formalizing a potential bias‐variance trade off associated with the use of additional factors as instruments, and developing a procedure that determines an optimal selection.

## Supporting information

Web Appendices referenced in Sections 1, 4, and 5 are available with this paper at the Biometrics website on Wiley Online Library, along with R code to our apply our methods.
Figure S1. Power results when testing the null hypothesis $H_{0}$ : θ_0_ = 0 for a 5% level test (*p* = 180 variants).
Figure S2. Power results when testing the null hypothesis $H_{0}$ : θ_0_ = 0 for a 5% level test (*p* = 90 variants).
Figure S3. Power results when testing the null hypothesis $H_{0}$ : θ_0_ = 0 for a 5% level test (*p* = 90 variants).
Figure S4. Power results when testing the null hypothesis $H_{0}$ : θ_0_ = 0 for a 5% level test (*p* = 360 variants).
Figure S5. Power results when testing the null hypothesis $H_{0}$ : θ_0_ = 0 for a 5% level test (*p* = 360 variants).
Figure S6. The performance of S‐LIML under conventional standard errors for *p* = 180 (Model 1).
Figure S7. RMSE results under correct specification (Model 1) for *p* = 180.
Figure S8. Root‐mean squared error (RMSE) results under locally invalid instruments (Model 3) for *p* = 180 and misspecification $\hat{r}\neq$ r.
Figure S9. Power results when testing the null hypothesis $H_{0}$ : θ_0_ = 0 for a 5% level test (*p* = 180 variants) under Model 5 in Section 4.3 and varied number of estimated factors *br* selected as instruments.
Figure S10. Coverage probabilities of confidence intervals under Model 1 and $H_{0}$ : $\theta_{0}=\theta_{0}$ for varying θ_0_.

Data S1

## Data Availability

The data that support the findings in this paper are openly available. Summary data on coronary artery disease from CARDIoGRAMplusC4D investigators were accessed at http://www.CARDIOGRAMPLUSC4D.ORG. Summary data on low‐density lipoprotein cholesterol concentrations from Neale Lab's analysis of UK Biobank data were accessed at http://www.nealelab.is/uk‐biobank/. Data on genetic variant correlations from The 1000 Genomes Project Consortium (1000 Genomes Project, Auton et al., 2015) were accessed using the twosampleMR R software package (Hemani et al., 2018).
