# Supplementary material for: Conditional inference in cis-Mendelian randomization using weak genetic factors
Source: Biometrics. Author manuscript; Available in PMC 2023 Dec 22. (PMC7615409; doi:10.1111/biom.13888)
Supplement: Supplementary Material [file EMS177415-supplement-Supplementary_Material.pdf]

SUPPORTING INFORMATION

Web Appendices referenced in Sections 1, 4, and 5 are available with this paper at the Biometrics website on Wiley Online Library, along with R code to our apply our methods.

Data S1

15410420, 0, Downloaded from https://onlinelibrary.wiley.com/doi/10.1111/biom.13888 by Test, Wiley Online Library on [25/06/2023]. See the Terms and Conditions (https://onlinelibrary.wiley.com/terms-and-conditions) on Wiley Online Library for rules of use; OA articles are governed by the applicable Creative Commons License
