# Supplementary material for: Conditional inference in cis‐Mendelian randomization using weak genetic factors
Source: Biometrics. 2023 Jun 19;79(4):3458–71. doi: 10.1111/biom.13888 (PMC7615409; doi:10.1111/biom.13888)
Supplement: Supplementary file 1 [file BIOM-79-3458-s001.pdf]

Supporting Information for "Conditional inference in *cis*-Mendelian randomization using weak genetic factors"

by Ashish Patel, Dipender Gill, Paul Newcombe, & Stephen Burgess

Web Appendix A Summary data

Web Appendix A.1 Available summary data

We work with two-sample summary data on univariable genetic variant–trait associations as commonly reported from genetic association studies. For any a-vector A, and b-vector B, let $\dot{var}(A)$ denote the $a \times a$ sample variance–covariance matrix of A, and $\dot{cov}(A, B)$ denote the $a \times b$ sample covariance matrix of A and B, that are calculated from an i.i.d. sample of size n_Y. Let $\ddot{var}(A)$ and $\ddot{cov}(A, B)$ denote analogous quantities calculated from an non-overlapping i.i.d. sample of size n_X. Let Y denote the outcome, X denote the exposure, and $Z = (Z_1, \dots, Z_p)'$ denote a p-vector genetic variants, where all variables are mean-centered.

First, for $j \in [p]$, we have summary data from univariable Y on Z_j linear regressions, $\hat{\beta}_{Y_j} = \dot{var}(Z_j)^{-1}\dot{cov}(Z_j, Y)$ and $\hat{\sigma}^2_{Y_j} = n_Y^{-1}\dot{var}(Z_j)^{-1}(\dot{var}(Y) - \dot{cov}(Z_j, Y)^2\dot{var}(Z_j)^{-1})$. Here, $\hat{\beta}_{Y_j}$ denotes the estimated association between Z_j and Y, and $\hat{\sigma}_{Y_j}$ the corresponding standard error. Similarly, $\hat{\beta}_{X_j} = \ddot{var}(Z_j)^{-1}\ddot{cov}(Z_j, X)$ is the estimated association between Z_j and X, and $\hat{\sigma}_{X_j}$ is the corresponding standard error, where $\hat{\sigma}^2_{X_j} = n_X^{-1}\ddot{var}(Z_j)^{-1}(\ddot{var}(X) - \ddot{cov}(Z_j, X)^2\ddot{var}(Z_j)^{-1})$. We further assume knowledge of the sample variances of the traits, $\ddot{var}(X)$ and $\dot{var}(Y)$, and knowledge of the pairwise correlation ρ_{jk} between any genetic variants Z_j and Z_k, $(j, k \in [p])$.

Let $\hat{\delta}_X = (\hat{\delta}_{X_1}, \dots, \hat{\delta}_{X_p})'$ and $\hat{\delta}_Y = (\hat{\delta}_{Y_1}, \dots, \hat{\delta}_{Y_p})'$ denote the vector of sample variant–trait covariances, and let $\delta_X = (\delta_{X_1}, \dots, \delta_{X_p})'$ and $\delta_Y = (\delta_{Y_1}, \dots, \delta_{Y_p})'$ denote their population counterparts. By similar arguments used in Wang and Kang (2021), we can recover the following relevant quantities from summary data:

1. variance-covariance matrix of genetic variants: $\dot{var}(Z)$ **and** $\ddot{var}(Z)$
Note that $\dot{v}_j \equiv (n_Y\hat{\sigma}^2_{Y_j} + \hat{\beta}^2_{Y_j})^{-1} = \dot{var}(Y)^{-1}\dot{var}(Z_j)$. Thus, $\dot{var}(Y)\rho_{jk}\sqrt{\dot{v}_j\dot{v}_k} = \dot{cov}(Z_j, Z_k)$. Therefore, we can construct the sample variance-covariance matrix of Z, $\dot{var}(Z)$. We can also compute $\ddot{var}(Z)$ by identical arguments.

2. genetic variant–trait covariances: $\hat{\delta}_X$ and $\hat{\delta}_Y$

Let $\dot{D}$ a diagonal matrix with its (j,j)-th element given by $v\dot{a}r(Z_j)$. Then, $\hat{\delta}_Y = \dot{D}\hat{\beta}_Y$. We can also compute $\hat{\delta}_X$ by identical arguments.

Web Appendix A.2 Approximation of log odds ratio estimates to linear regression coefficients

Here we describe the procedure for obtaining estimates of $\hat{\delta}_{Y_j} = c\dot{o}v(Y, Z_j)$ from available summary data on log odds ratio estimates from univariable Y on Z_j logit regression coefficients $\hat{\beta}_{Y_j}$, and corresponding standard errors $\hat{\sigma}_{Y_j}$. The procedure relies on individual variants Z_j explaining a low proportion of variation in the outcome Y, which is plausible in typical MR applications. The standardized least squares estimate of a linear regression of Y on Z_j is approximately $\mathcal{Z}_j = n_Y^{-\frac{1}{2}}\hat{\sigma}_{Y_j}^{-1}\hat{\beta}_{Y_j}$. Thus, $\hat{\delta}_{Y_j} = \mathcal{Z}_j\sqrt{v\dot{a}r(Z_j)}\sqrt{v\dot{a}r(Y)}$. For other summary data methods which use estimated coefficients of univariable linear regressions as inputs, we can compute $\bar{\beta}_{Y_j} = v\dot{a}r(Z_j)^{-1}\hat{\delta}_{Y_j}$ for approximated effect size estimates, and the approximated standard errors based on equating z-scores are $\bar{\sigma}_{Y_j} = \bar{\beta}_{Y_j}\hat{\sigma}_{Y_j}\hat{\beta}_{Y_j}^{-1}$.

Web Appendix A.3 Individual-level data model

Let Y denote a continuous outcome, X a continuous exposure, and $Z = (Z_1, \ldots, Z_p)'$ a p-vector of mean-centered genetic variants. Our summary-data model can be motivated from the following individual-level data model

$$\begin{aligned} Y &= \alpha_0 + X\theta_0 + U \\ X &= \gamma_0 + (\Lambda' Z)'\gamma_1 + V, \end{aligned}$$

where $E[U|Z] = 0$, $E[U^2|\Lambda' Z] = \sigma_U^2$, $E[V|Z] = 0$, and $E[V|\Lambda' Z] = \sigma_V^2$. This means that the genetic variants Z are valid instruments, and that when using the linear combination of variants $\Lambda' Z$ as instruments, the errors are homoscedastic. Note that the reduced form model for Y can be written

$$Y = \phi_0 + (\Lambda' Z)'\phi_1 + \epsilon,$$

where $E[\epsilon|Z] = 0$, $E[\epsilon^2|\Lambda' Z] = \sigma_\epsilon^2$, $\phi_0 = \alpha_0 + \theta_0\gamma_0$, $\phi_1 = \gamma_1\theta_0$, and $\epsilon = U + \theta_0 V$. Therefore, given an i.i.d. n_X-sample on (X, Z), we have $E[\Lambda'\hat{\delta}_X] = \Lambda' E[ZX] = \Lambda'\delta_X$, and $var(\Lambda'\hat{\delta}_X) = E[(\Lambda' Z)(\Lambda' Z)' \cdot var(X|\Lambda' Z)]n_X^{-1} = \Omega_0\sigma_V^2 n_X^{-1}$. The mean and variance of $\Lambda'\hat{\delta}_Y$ from an non-overlapping i.i.d. n_Y-sample on (Y, Z) can be calculated by identical arguments. Hence, a central limit theorem motivates Assumption 2.2.

Web Appendix B Preparatory lemmata

We use the following abbreviations: 'T' denotes the triangle inequailty; 'CH' denotes Chebyshev's inequality; 'CS' denotes the Cauchy-Schwarz inequality; 'M' denotes the Markov inequality; 'w.p.a.1' denotes 'with probability approaching 1'; 'RHS' denotes 'right-hand side'; 'LHS denotes 'left-hand side'. For any vector or matrix A, $\|A\|$ denotes the Euclidean norm.

Web Appendix B.1 Bai (2003)'s results on factor loadings

Let n_Z denote the sample size of the reference sample used to compute the genetic variant correlation matrix ρ. The reference sample is permitted to overlap with one of the genetic association studies. We further assume that n_Z is $\Theta(n)$ so that, in our asymptotic analysis, we consider the sample sizes of the genetic association studies (n_X and n_Y) and any reference sample (n_Z) to all be increasing at the same rate with the number of genetic variants p.

Let z_{ki} denote the mean-centered $k-$th genetic variant for the i-th individual, $i \in [n_Z]$. Let $z_i = (z_{1i}, \ldots, z_{pi})'$, and let $z = (z_1, \ldots, z_{n_Z})'$ denote the $n_Z \times p$ matrix of mean-centered genotypes. Under Bai and Ng (2002)'s approximate factor model, we have

$$z = F\Lambda' + e,$$

where $\Lambda = (\lambda_1, \ldots, \lambda_p)'$ denotes a $p \times r$ matrix of factor loadings, $F = (F_1, \ldots, F_{n_Z})'$ denotes an $n_Z \times r$ matrix of factors, and $e = (e_1, \ldots, e_{n_Z})'$ denotes a $n_Z \times p$ matrix of idiosyncratic errors.

Let the columns of $\bar{\Lambda}$ denote the first r eigenvectors of the $p \times p$ sample variance-covariance matrix $\widehat{var}(Z) = z'z/n_Z$ multiplied by $\sqrt{p}$. Let $\bar{F} = (z\bar{\Lambda})(\bar{\Lambda}'\bar{\Lambda})^{-1}$, and $\hat{F} = \bar{F}(\bar{F}'\bar{F}/n_Z)^{-\frac{1}{2}}$. Then, $\hat{F}'\hat{F}/n_Z = I_r$, and $\hat{\Lambda}' = \hat{F}'z/n_Z = (\bar{F}'\bar{F}/n_Z)^{-\frac{1}{2}}(\bar{\Lambda}'\bar{\Lambda})^{-1}(\bar{\Lambda}'z'z/n_Z)$.

Let $H = (\Lambda'\Lambda/p)(F'\hat{F}/n_Z)D_{np}^{-1}$ where D_{np} is an $r \times r$ diagonal matrix with its diagonal entries equal to the first r largest eigenvalues of $(zz'/(n_Z p))$ in decreasing order.

Lemma 1. As $n, p \to \infty$, **(i)** $\|H\| = O_P(1)$; **(ii)** $\|H^{-1}\| = O_P(1)$.

Proof. By Lemma A.3(i) of Bai (2003, p.161), $D_{np} \xrightarrow{P} D_0$, a diagonal matrix consisting of the eigenvalues of $\Sigma_\Lambda \Sigma_F$. Hence, w.p.a.1, D_{np}^{-1} exists since Σ_Λ and Σ_F are positive definite, and $\|D_{np}^{-1}\| = O_P(1)$. By construction, $\hat{F}'\hat{F}/n_Z = I_r$, so that $\|\hat{F}'\hat{F}/n_Z\| = O_P(1)$. By CS, Assumption 1(iv), and M, $\|F'F/n_Z\| \leq \sum_{i=1}^{n_Z} \|F_i\|^2/n_Z = O_P(1)$. Similarly, for all $k \in [p]$, by Assumption 1(v), $\|\lambda_k\| \leq C_\lambda < \infty$. Therefore, $\|\Lambda'\Lambda/p\| \leq \sum_{k=1}^{p} \|\lambda_k\|^2/p = O(1)$. Also, by CS, $\|H\| \leq \|\Lambda'\Lambda/p\| \cdot \|F'F/n_Z\|^{\frac{1}{2}} \cdot \|\hat{F}'\hat{F}/n_Z\|^{\frac{1}{2}} \cdot \|D_{np}^{-1}\|$, so that $\|H\| = O_P(1)$. H is invertible; see Bai (2003, p.145). Therefore, by Part (i), $\|H^{-1}\| = O_P(1)$. $\square$

Lemma 2. As $n, p \to \infty$, $\|\hat{\lambda}_k - H^{-1}\lambda_k\| = O_P(n^{-\frac{1}{2}}) + O_P(\min(n, p)^{-1})$, $k \in [p]$.

Proof. By Bai (2003, p.165), for any $k \in [p]$, we have

$$\hat{\lambda}_k - H^{-1}\lambda_k = H' \frac{1}{n_Z} \sum_{i=1}^{n_Z} F_i e_{ki} + \frac{1}{n_Z} \sum_{i=1}^{n_Z} \hat{F}_i (F_i - H^{-1\prime} \hat{F}_i)' \lambda_k + \frac{1}{n_Z} \sum_{i=1}^{n_Z} (\hat{F}_i - H' F_i) e_{ki}, \tag{S.1}$$

where e_{ki} is the (i,k)-th element of the matrix of idiosyncratic errors e. Hence, by CS,

$$\|\hat{\lambda}_k - H^{-1}\lambda_k\| = \frac{1}{\sqrt{n_Z}} \|H\| \cdot \Big\| \frac{1}{\sqrt{n_Z}} \sum_{i=1}^{n_Z} F_i e_{ki} \Big\| + \Big\| \frac{1}{n_Z} \sum_{i=1}^{n_Z} \hat{F}_i (F_i - H'^{-1} \hat{F}_i)' \Big\| \cdot \|\lambda_k\| + \Big\| \frac{1}{n_Z} \sum_{i=1}^{n_Z} (\hat{F}_i - H' F_i) e_{ki} \Big\|,$$

where the first term on the RHS is $O_P(n^{-\frac{1}{2}})$ by Lemma 1(i), Assumption D of Bai (2003, p.141), and M. The second term on the RHS is $O_P(\min(n,p)^{-1})$ by Lemma B.3 of Bai (2003, p.165), Lemma 1(ii), and Assumption 1(v). The third term on the RHS is $O_P(\min(n,p)^{-1})$ by Lemma B.1 of Bai (2003, p.163). $\square$

Web Appendix B.2 Weak dependence of model errors

Under our model assumptions, $\delta_Y = \delta_X \theta_0$. We can write $\hat{\delta}_Y - \hat{\delta}_X \theta_0 = \varepsilon_Y - \varepsilon_X \theta_0$, where $\varepsilon_X = \hat{\delta}_X - \delta_X$ and $\varepsilon_Y = \hat{\delta}_Y - \delta_Y$. Let $\varepsilon = \varepsilon_Y - \theta_0 \varepsilon_X$, and define $\varepsilon^\star_{X_k} = \sqrt{n} \varepsilon_{X_k}$ and $\varepsilon^\star_k = \sqrt{n} \varepsilon_k$, $k \in [p]$. Under an assumption of random sampling, and $E[\varepsilon^{\star 2}_{X_k}] < \infty$, $E[\varepsilon^{\star 2}_k] < \infty$, the re-scaled errors are bounded in probability. We maintain the following assumptions which are analogous to Assumptions A and B in Bai and Ng (2010, pp.1581-2), and they ensure the sampling errors from estimating the space spanned by the factor loadings are negligible as $n, p \to \infty$.

Assumption S0. There exists a universal positive constant C, such that, as $n_Z, n, p \to \infty$, **(i)** for each $i \in [n_Z]$, $\sum_{k=1}^p |\mathbb{E}[e_{ki} \varepsilon^\star_k]| \leq C$ and $\sum_{k=1}^p |\mathbb{E}[e_{ki} \varepsilon^\star_{X_k}]| \leq C$; **(ii)** $\mathbb{E}\big[\| \frac{1}{\sqrt{n_Z p}} \sum_{i=1}^{n_Z} \sum_{k=1}^p F_i (e_{ki} \varepsilon^\star_k - \mathbb{E}[e_{ki} \varepsilon^\star_k]) \|\big] \leq C$ and $\mathbb{E}\big[\| \frac{1}{\sqrt{n_Z p}} \sum_{i=1}^{n_Z} \sum_{k=1}^p F_i (e_{ki} \varepsilon^\star_{X_k} - \mathbb{E}[e_{ki} \varepsilon^\star_{X_k}]) \|\big] \leq C$; **(iii)** for each $i \in [n_Z]$, $\mathbb{E}\big[| \frac{1}{\sqrt{p}} \sum_{k=1}^p (e_{ki} \varepsilon^\star_k - \mathbb{E}[e_{ki} \varepsilon^\star_k]) |^2\big] \leq C$ and $\mathbb{E}\big[| \frac{1}{\sqrt{p}} \sum_{k=1}^p (e_{ki} \varepsilon^\star_{X_k} - \mathbb{E}[e_{ki} \varepsilon^\star_{X_k}]) |^2\big] \leq C$; **(iv)** for each $i \in [n_Z]$, $\mathbb{E}\big[| \frac{1}{\sqrt{p}} \sum_{k=1}^p e_{ki} |^2\big] \leq C$ and $\mathbb{E}\big[\| \frac{1}{\sqrt{n_Z p}} \sum_{i=1}^{n_Z} \sum_{k=1}^p F_i e_{ki} \|\big] \leq C$.

Assumptions S0(i)–(iii) restrict the extent to which idiosyncratic errors of the approximate factor model can be correlated with sampling errors of genetic associations in the linear model. These assumptions are satisfied when a separate reference sample is used only to estimate the variance-covariance matrix of genetic variants, $var(Z)$. In general, these assumptions are also likely to hold when the latent factors explain a large proportion of genetic variation. Conversely, Assumptions S0(i)–(iii) may be violated when variants are in less structured linkage disequilibrium, and when the structural linear model used to describe genetic variant–trait associations is misspecified. For example, this could occur if there is selection bias from the recruitment of study participants having survived competing risks to exposure and outcome (Schooling et al., 2021). For Parts (ii)–(iv) of Assumption S0, we note that these are conditions on zero-mean sums, and are analogous to Assumptions C5, D, F1, and F2 of Bai (2003, p.141 and p.144).

Web Appendix B.3 Covariance terms

Lemma 3. As $n, p \to \infty$, **(i)** $\Omega_0 = O_P(p)$; **(ii)** $\Omega_0^{-1} = O_P(p^{-1})$; **(iii)** $\hat{\Omega}_0 - H^{-1}\Omega_0 H^{-1\prime} = o_P(p)$; **(iv)** $\hat{\Omega}_0 = O_P(p)$; **(v)** $\hat{\Omega}_0^{-1} = O_P(p^{-1})$; **(vi)** $\hat{\Omega}_0^{-1} - H'\Omega_0^{-1}H = o_P(p^{-1})$.

Proof. First, for Parts (i) and (ii), $\Omega_0 = O(\Lambda' var(Z)\Lambda) = O(p)$ since $\Lambda'\Lambda = \Theta(p)$, $cov(Z_j, Z_k) = \Theta(1)$ for all $j, k \in [p]$, and Ω_0 is invertible. For Part (iii), note that we can write $\hat{\Lambda}' v\hat{a}r(Z)\hat{\Lambda} - H^{-1}\Lambda' var(Z)\Lambda H^{-1\prime} = R_1 + 2R_2 + R_3$, where $R_1 = (\hat{\Lambda} - \Lambda H^{-1\prime})' v\hat{a}r(Z)(\hat{\Lambda} - \Lambda H^{-1\prime})$, $R_2 = H^{-1}\Lambda' v\hat{a}r(Z)(\hat{\Lambda} - \Lambda H^{-1\prime})$, and $R_3 = H^{-1}\Lambda'(v\hat{a}r(Z) - var(Z))\Lambda H^{-1\prime}$. Note that $R_1 = O((\hat{\Lambda} - \Lambda H^{-1\prime})'(\hat{\Lambda} - \Lambda H^{-1\prime}))$, since $c\hat{o}v(Z_j, Z_k) = \Theta(1)$. Therefore, $\|R_1\| \leq \sum_{k=1}^{p} \|\hat{\lambda}_k - H^{-1}\lambda_k\|^2 = O_P(n^{-1}p) + O_P(\min(n,p)^{-2}p)$ by Lemma 2. Similarly, we have $\|R_2\| \leq \|\hat{\Lambda} - \Lambda H^{-1\prime}\| \cdot \|\Lambda H^{-1}\|$. By CS and Lemma 2, $\|\hat{\Lambda} - \Lambda H^{-1\prime}\|^2 \leq (\hat{\Lambda} - \Lambda H^{-1\prime})'(\hat{\Lambda} - \Lambda H^{-1\prime}) \leq \sum_{k=1}^{p} \|\hat{\lambda}_k - H^{-1}\lambda_k\|^2 = O_P(n^{-1}p) + O_P(\min(n,p)^{-2}p)$, and $\|\Lambda H^{-1}\|^2 \leq \|H^{-1}\|^2 \cdot \|\Lambda'\Lambda\| = O_P(p)$ by Lemma 1(ii) and Assumption 1(v). Hence, by CS, $\|R_2\| = O_P(n^{-\frac{1}{2}}p) + O_P(\min(n,p)^{-1}p)$. For R_3, we have that $H^{-1}p^{-1}\Lambda'(v\hat{a}r(Z) - var(Z))\Lambda H^{-1\prime} = o_P(1)$ since for any $j, k \in [p]$, $c\hat{o}v(Z_j, Z_k) - cov(Z_j, Z_k) = o_P(1)$ by the weak law of large numbers. Hence, $\|R_3\| = o_P(p)$. For Part (iv), note that by T and CS, $\|\hat{\Omega}_0\| \leq \|\hat{\Omega}_0 - H^{-1}\Omega_0 H^{-1\prime}\| + \|H^{-1}\|^2\|\Omega_0\| = O_P(p)$ by Lemma 1(ii), and Parts (i) and (iii). For Part (v), by Part (iii), $p^{-1}(\hat{\Omega}_0 - H^{-1}\Omega_0 H^{-1\prime}) = o_P(1)$, and $p^{-1}H^{-1}\Omega_0 H^{-1\prime}$ is invertible since H^{-1} is invertible, and Ω_0 is invertible. Hence, $p^{-1}\hat{\Omega}_0$ is invertible w.p.a.1, and $\hat{\Omega}_0^{-1} = O_P(p)$ by Parts (ii) and (iv). For (vi), we can write $\hat{\Omega}_0^{-1} - H'\Omega_0^{-1}H = \hat{\Omega}_0^{-1}(H^{-1}\Omega_0 H^{-1\prime} - \hat{\Omega}_0)H'\Omega_0^{-1}H$, so that by CS, $\|\hat{\Omega}_0^{-1} - H'\Omega_0^{-1}H\| \leq \|\hat{\Omega}_0^{-1}\| \cdot \|\hat{\Omega}_0 - H^{-1}\Omega_0 H^{-1\prime}\| \cdot \|H\|^2\|\Omega_0^{-1}\| = o_P(p^{-1})$ by Lemma 1(i), and Parts (ii), (iii), and (v). □

Web Appendix B.4 Derivative terms

Let $\hat{G} = -\hat{\Lambda}'\hat{\delta}_X$, $\bar{G} = -H^{-1}\Lambda'\hat{\delta}_X$, and $G = -H^{-1}\Lambda'\delta_X$. Similarly, let $\hat{G}_Y = -\hat{\Lambda}'\hat{\delta}_Y$, $\bar{G}_Y = -H^{-1}\Lambda'\hat{\delta}_Y$, and $G_Y = -H^{-1}\Lambda'\delta_Y$.

Lemma 4. As $n, p \to \infty$, **(i)** $\hat{G} - \bar{G} = O_P(1) + o_P(n^{-\frac{1}{2}}p^{\frac{1}{2}})$; **(ii)** $\bar{G} - G = O_P(n^{-\frac{1}{2}}p^{\frac{1}{2}})$; **(iii)** $G = O_P(p^{\frac{1}{2}})$ and $\hat{G} = O_P(p^{\frac{1}{2}})$.

Proof. For Part (i), note that $\hat{G} - \bar{G} = -\sum_{k=1}^{p}(\hat{\lambda}_k - H^{-1}\lambda_k)\delta_X - \sum_{k=1}^{p}(\hat{\lambda}_k - H^{-1}\lambda_k)\varepsilon_{X_k}$. The first term on the RHS is bounded by $(\sum_{k=1}^{p}\|\hat{\lambda}_k - H^{-1}\lambda_k\|^2)^{\frac{1}{2}}\|\delta_X\| = O_P(1)$ by Assumption 3, Lemma 2, and CS, and the second term on the RHS is $o_P(n^{-\frac{1}{2}}p^{-\frac{1}{2}})$ by identical arguments used in Proof of Lemma 8(i) below. For Part (ii), by Assumption 2 and CH, $\Lambda'(\hat{\delta}_X - \delta_X) = O_P(n^{-\frac{1}{2}}p^{\frac{1}{2}})$, so that by Lemma 1(ii), $\bar{G} - G = O_P(n^{-\frac{1}{2}}p^{\frac{1}{2}})$. For Part (iii), by CS, $\|G\| \leq \|H^{-1}\| \cdot \|\Lambda'\Lambda\|^{\frac{1}{2}}\|\delta_X\| = O_P(p^{\frac{1}{2}})$ by Lemma 1(ii) and Assumptions 1(v) and 3. The final result of Part (iii) then follows by Parts (i), (ii), and T. □

Lemma 5. As $n, p \to \infty$ **(i)** $\hat{G}_Y - \bar{G}_Y = O_P(1) + o_P(n^{-\frac{1}{2}}p^{\frac{1}{2}})$; **(ii)** $\bar{G}_Y - G_Y = O_P(n^{-\frac{1}{2}}p^{\frac{1}{2}})$; **(iii)** $G_Y = O_P(p^{\frac{1}{2}})$ and $\hat{G}_Y = O_P(p^{\frac{1}{2}})$.

Proof. The results follow by analogous arguments used in the Proof of Lemma 4, where here we also note that $\delta_Y = \delta_X\theta_0$, so that $\|\delta_X\| = O(1)$ implies that $\|\delta_Y\| = O(1)$. □

Covariance terms II

Let $\Omega(\theta) = H^{-1}\Omega_0 H^{-1\prime} \cdot (n_Y^{-1}\sigma_\epsilon^2 + \theta^2 n_X^{-1}\sigma_V^2)$, $\Omega = \Omega(\theta_0)$, and for any consistent estimator $\dot\theta$ for θ_0, let $\hat\Omega(\dot\theta) = \hat\Omega_0 \cdot (n_Y^{-1}\hat\sigma_\epsilon^2 + \dot\theta^2 n_X^{-1}\hat\sigma_V^2)$.

Lemma 6. For $n, p \to \infty$, **(i)** $\hat\sigma_\epsilon^2 - \sigma_\epsilon^2 = o_P(1)$; **(ii)** $\hat\sigma_V^2 - \sigma_V^2 = o_P(1)$.

Proof. For Part (i), note that

$$\begin{aligned}
\hat\sigma_\epsilon^2 &= v\dot{a}r(Y) - \hat G_Y' \hat\Omega_0^{-1}\hat G_Y \\
&= var(Y) + (v\dot{a}r(Y) - var(Y)) - (\hat G_Y - G_Y)'\hat\Omega_0^{-1}\hat G_Y - G_Y'(\hat\Omega_0^{-1} - H'\Omega_0^{-1}H)\hat G_Y \\
&\quad - G_Y' H'\Omega_0^{-1}H(\hat G_Y - G_Y) - G_Y' H'\Omega_0^{-1} H G_Y \\
&= var(Y) + o_P(1) + (O_P(n^{-\frac{1}{2}}p^{\frac{1}{2}}) + O_P(1))O_P(p^{-1})O_P(p^{\frac{1}{2}}) + O_P(p)o_P(p^{-1}) \\
&\quad + O_P(p^{\frac{1}{2}})O_P(p^{-1})(O_P(n^{-\frac{1}{2}}p^{\frac{1}{2}}) + O_P(1)) - (\Lambda'\delta_Y)'H^{-1\prime}H'\Omega_0^{-1}HH^{-1}(\Lambda'\delta_Y) \\
&= var(Y) - (\Lambda'\delta_Y)'\Omega_0^{-1}(\Lambda'\delta_Y) + o_P(1) + O_P(n^{-\frac{1}{2}}) + O_P(p^{-\frac{1}{2}}) \\
&= \sigma_\epsilon^2 + o_P(1),
\end{aligned}$$

by consistency of $v\dot{a}r(Y)$ for $var(Y)$, Lemmas 1(i), 3(ii), 3(v), 3(vi), and 5, and T. Part (ii) follows by identical arguments. □

Lemma 7. For any consistent estimator $\dot\theta$ of θ_0, as $n, p \to \infty$, **(i)** $\Omega = O_P(n^{-1}p)$; **(ii)** $\Omega^{-1} = O_P(np^{-1})$; **(iii)** $\hat\Omega(\dot\theta) - \Omega = o_P(n^{-1}p)$; **(iv)** $\hat\Omega(\dot\theta) = O_P(n^{-1}p)$; **(v)** $\hat\Omega(\dot\theta)^{-1} = O_P(np^{-1})$; **(vi)** $\hat\Omega(\dot\theta)^{-1} - \Omega^{-1} = o_P(np^{-1})$.

Proof. First, $\Omega = O_P(n^{-1}p)$ by Lemmas 1(ii), 3(i), and CS. Similarly, $\Omega^{-1} = H'\Omega_0^{-1}H \cdot (n_Y^{-1}\sigma_\epsilon^2 + \theta_0^2 n_X^{-1}\sigma_V^2)^{-1} = O_P(np^{-1})$ by Lemmas 1(i), 3(ii), and CS. For Part (iii), note that

$$\begin{aligned}
\hat\Omega(\dot\theta) - \Omega &= (\hat\Omega_0 - H^{-1}\Omega_0 H^{-1\prime}) \cdot (n_Y^{-1}\hat\sigma_\epsilon^2 + \dot\theta^2 n_X^{-1}\hat\sigma_V^2) \\
&\quad + H^{-1}\Omega_0 H^{-1\prime} \cdot \big(n_Y^{-1}(\hat\sigma_\epsilon^2 - \sigma_\epsilon^2) + \dot\theta^2 n_X^{-1}(\hat\sigma_V^2 - \sigma_V^2) + (\dot\theta - \theta_0)(\dot\theta + \theta_0)n_X^{-1}\sigma_V^2\big) \\
&= o_P(p)O_P(n^{-1}) + o_P(p)O_P(n^{-1}) + O_p(p)\big(o_P(n^{-1}) + o_P(n^{-1}) + o_P(n^{-1})\big) \\
&= o_P(n^{-1}p),
\end{aligned}$$

where the second equality follows by Lemmas 1(ii), 3(i), (iii), 6, and M. Part (iv) then follows by Parts (i), (iii), and T. For Part (v), note that Part (iii) means that $pn^{-1}(\hat\Omega(\dot\theta) - \Omega) = o_P(1)$, and $p^{-1}n\Omega$ is invertible, so that $p^{-1}n\hat\Omega(\dot\theta)$ is invertible w.p.a.1, and $\hat\Omega(\dot\theta)^{-1} = O_P(np^{-1})$. Finally, for Part (vi), note that we can write $\hat\Omega(\dot\theta)^{-1} - \Omega^{-1} = \hat\Omega(\dot\theta)^{-1}(\Omega - \hat\Omega(\dot\theta))\Omega^{-1} = O_P(n^2p^{-2})o_P(n^{-1}p) = o_P(np^{-1})$ by Parts (ii), (iii), and (v). □

Web Appendix B.5 Residual term

Let $\hat g(\theta) = \hat\Lambda'(\hat\delta_Y - \hat\delta_X\theta)$, and $g(\theta) = H^{-1}\Lambda'(\hat\delta_Y - \hat\delta_X\theta)$.

Lemma 8. As $n, p \to \infty$, **(i)** $\hat{g}(\theta_0) - g(\theta_0) = o_P(n^{-\frac{1}{2}} p^{\frac{1}{2}})$; **(ii)** $\hat{g}(\theta_0) = O_P(n^{-\frac{1}{2}} p^{\frac{1}{2}})$; **(iii)** $\hat{g}(\dot{\theta}) = o_P(p^{\frac{1}{2}})$ for any $\dot{\theta} \xrightarrow{P} \theta_0$.

Proof. For Part (i), we can write

$$\begin{aligned}
\hat{g}(\theta_0) - g(\theta_0) &= \sum_{k=1}^{p} (\hat{\lambda}_k - H^{-1}\lambda_k)\varepsilon_k \\
&= H' \frac{1}{n_Z} \sum_{i=1}^{n_Z} \sum_{k=1}^{p} F_i e_{ki} \varepsilon_k + \frac{1}{n_Z} \sum_{i=1}^{n_Z} \sum_{k=1}^{p} \hat{F}_i (F_i - H^{-1\prime} \hat{F}_i)' \lambda_k \varepsilon_k + \frac{1}{n_Z} \sum_{i=1}^{n_Z} \sum_{k=1}^{p} (\hat{F}_i - H' F_i) e_{ki} \varepsilon_k \\
&:= H' \mathcal{R}_1 + \mathcal{R}_2 + \mathcal{R}_3,
\end{aligned}$$

where the second equality follows by Equation (S.1). First, note that

$$\sqrt{n} \mathcal{R}_1 = \sqrt{\frac{p}{n_Z}} \sqrt{\frac{1}{n_Z p}} \sum_{k=1}^{p} \sum_{i=1}^{n_Z} F_i (e_{ki} \varepsilon_k^\star - \mathbb{E}[e_{ki} \varepsilon_k^\star]) + \frac{1}{n_Z} \sum_{i=1}^{n_Z} F_i \sum_{k=1}^{p} \mathbb{E}[e_{ki} \varepsilon_k^\star].$$

By CS,

$$\begin{aligned}
\sqrt{n} \|\mathcal{R}_1\| &\leq \sqrt{\frac{p}{n_Z}} \Big\| \frac{1}{\sqrt{n_Z p}} \sum_{k=1}^{p} \sum_{i=1}^{n_Z} F_i (e_{ki} \varepsilon_k^\star - \mathbb{E}[e_{ki} \varepsilon_k^\star]) \Big\| + \Big(\frac{1}{n_Z} \sum_{i=1}^{n_Z} \|F_i\|^2 \Big)^{\frac{1}{2}} \Big(\frac{1}{n_Z} \sum_{i=1}^{n_Z} \Big(\sum_{k=1}^{p} |\mathbb{E}[e_{ki} \varepsilon_k^\star]| \Big)^2 \Big)^{\frac{1}{2}} \\
&= O_P(n^{-\frac{1}{2}} p^{\frac{1}{2}}) + O_P(1),
\end{aligned}$$

by Assumption 1(iv), Assumptions S0(i) and (ii), CS, T, and M. Thus, by Lemma 1(i) and CS, $H' \mathcal{R}_1 = O_P(n^{-1} p^{\frac{1}{2}}) + O_P(n^{-\frac{1}{2}}) = o_P(n^{-\frac{1}{2}} p^{\frac{1}{2}})$.

For $\mathcal{R}_2$, by CS, Lemma 1(ii), and Lemma B.3 of Bai (2003, p.165),

$$\begin{aligned}
\Big\| \frac{1}{n_Z} \hat{F}' (F - \hat{F} H^{-1}) \Big\| &\leq \Big\| \frac{1}{n_Z} \hat{F}' (F H - \hat{F}) \Big\| \cdot \|H^{-1}\| \\
&= O_P(\min(n, p)^{-1}).
\end{aligned}$$

Also, $\|\Lambda' \varepsilon\| = O_P(p^{\frac{1}{2}} n^{-\frac{1}{2}})$ by Assumption 2. Hence, by CS, $\|\mathcal{R}_2\| = O_P(p^{\frac{1}{2}} n^{-\frac{1}{2}}) \times O_P(\min(n, p)^{-1}) = o_P(n^{-\frac{1}{2}} p^{\frac{1}{2}})$.

Finally, for $\mathcal{R}_3$, note that

$$\sqrt{n} \mathcal{R}_3 = \frac{1}{n_Z} \sum_{i=1}^{n_Z} (\hat{F}_i - H' F_i) \sum_{k=1}^{p} (e_{ki} \varepsilon_k^\star - \mathbb{E}[e_{ki} \varepsilon_k^\star]) + \frac{1}{n_Z} \sum_{i=1}^{n_Z} (\hat{F}_i - H' F_i) \sum_{k=1}^{p} \mathbb{E}[e_{ki} \varepsilon_k^\star].$$

Then, by CS,

$$
\begin{aligned}
\|\mathcal{R}_3\| &\le \sqrt{\frac{p}{n}}\Big(\frac{1}{n_Z}\sum_{i=1}^{n_Z}\|\hat{F}_i - H'F_i\|^2\Big)^{\frac{1}{2}}\Big(\frac{1}{n_Z}\sum_{i=1}^{n_Z}\Big|\frac{1}{\sqrt{p}}\sum_{k=1}^{p}(e_{ki}\varepsilon_k^\star - \mathbb{E}[e_{ki}\varepsilon_k^\star])\Big|^2\Big)^{\frac{1}{2}} \\
&\quad + \frac{1}{\sqrt{n}}\Big(\frac{1}{n_Z}\sum_{i=1}^{n_Z}\|\hat{F}_i - H'F_i\|^2\Big)^{\frac{1}{2}}\Big(\frac{1}{n_Z}\sum_{i=1}^{n_Z}\big(\sum_{k=1}^{p}|\mathbb{E}[e_{ki}\varepsilon_k^\star]|\big)^2\Big)^{\frac{1}{2}} \\
&= O_P(n^{-\frac{1}{2}}p^{\frac{1}{2}}\cdot \min(n,p)^{-\frac{1}{2}}) + O_P(n^{-\frac{1}{2}}) \\
&= o_P(n^{-\frac{1}{2}}p^{\frac{1}{2}}),
\end{aligned}
$$

by M, T, Lemma A.1 of Bai (2003, p.159), and Assumptions S0(i) and (iii). By the above results on the remainder terms $\mathcal{R}_1$, $\mathcal{R}_2$, and $\mathcal{R}_3$, Part (i) follows by T.

For Part (ii), note that $g(\theta_0) = O_P(n^{-\frac{1}{2}}p^{\frac{1}{2}})$ by Lemma 1(ii), Assumption 2, and CH. Hence, by Part (i) and T, $\hat{g}(\theta_0) = O_P(n^{-\frac{1}{2}}p^{\frac{1}{2}})$. Similarly, $\hat{g}(\dot{\theta}) - \hat{g}(\theta_0) = \hat{G}(\dot{\theta} - \theta_0) = o_P(p^{\frac{1}{2}})$ by Lemma 4(iii), CS, and consistency of $\dot{\theta}$ for θ_0. Hence, Part (iii) follows by Part (ii) and T. □

Web Appendix C Proof of main results

Web Appendix C.1 Proof of Theorem 1

The F-LIML estimator is given by $\hat{\theta}_F = \arg\max_\theta \hat{Q}(\theta)$, where $\hat{Q}(\theta) = -\hat{g}(\theta)'\hat{\Omega}(\theta)^{-1}\hat{g}(\theta)/2$. Under the conditions of Theorem 1, the estimator $\hat{\theta}_F$ can be shown to be consistent for θ_0 by applying standard arguments used to establish consistency of extremum estimators; see, for example, Newey and McFadden (1994, Theorem 2.1, p.2121) and Zhao et al. (2020, Proof of Theorem 3.1). By a first-order Taylor expansion, there exists a $\dot{\theta}$ in the line segment joining $\hat{\theta}_F$ and θ_0 such that $\nabla_{\theta\theta}\hat{Q}(\dot{\theta})(\hat{\theta}_F - \theta_0) = -\nabla_\theta\hat{Q}(\theta_0)$. Dividing both sides by $-G'\Omega^{-1}G$, and by Lemmas 10(ii) and (iii) below, it follows that $(G'\Omega^{-1}G)^{\frac{1}{2}}(\hat{\theta}_F - \theta_0) \xrightarrow{D} N(0,1)$. Let $\hat{\Omega} = \hat{\Omega}(\hat{\theta}_F)$. Now, since $\hat{G}'\hat{\Omega}^{-1}\hat{G} - G'\Omega^{-1}G = \hat{G}'(\hat{\Omega}^{-1} - \Omega^{-1})\hat{G} + (\hat{G} - G)'\Omega^{-1}\hat{G} + G'\Omega^{-1}(\hat{G} - G) = o_P(n)$ by identical arguments used in the Proof of Lemma 9(ii) below. Therefore, $(G'\Omega^{-1}G)^{-1}(\hat{G}'\hat{\Omega}^{-1}\hat{G}) = o_P(1)$, and the result of the theorem follows by Slutsky's lemma. □

Lemma 9. Under Assumptions 1–3, and Equations (1)–(2), as $n, p \to \infty$, **(i)** $\nabla_\theta\hat{Q}(\theta_0) = -G'\Omega^{-1}\hat{g}(\theta_0) + o_P(n^{-\frac{1}{2}})$; **(ii)** $\nabla_{\theta\theta}\hat{Q}(\dot{\theta}) = -G'\Omega^{-1}G + o_P(n)$.

Proof. For Part (i), we have

$$
\begin{aligned}
\nabla_\theta\hat{Q}(\theta_0) &= -\hat{G}'\hat{\Omega}^{-1}\hat{g}(\theta_0) + \theta_0 n_X^{-1}\hat{\sigma}_V^2\hat{g}(\theta_0)'\hat{\Omega}_0\hat{\Omega}^{-2}\hat{g}(\theta_0) \\
&= -(\hat{G} - G)'\hat{\Omega}^{-1}\hat{g}(\theta_0) - G'(\hat{\Omega}^{-1} - \Omega^{-1})\hat{g}(\theta_0) - G'\Omega^{-1}\hat{g}(\theta_0) + O_P(1) \\
&= -G'\Omega^{-1}\hat{g}(\theta_0) + O_P(1) + O_P(n^{\frac{1}{2}}p^{-\frac{1}{2}}) + o_P(n^{\frac{1}{2}}) \\
&= -G'\Omega^{-1}\hat{g}(\theta_0) + o_P(n^{\frac{1}{2}}),
\end{aligned}
$$

where the second equality follows by Lemmas 3(iv), 6(ii), 7(v), 8(ii), and M, and the third equality follows by Lemmas 4(i), 4(ii), 4(iii), 7(v), 7(vi), and 8(ii).

Similarly, for Part (ii),

$$\begin{aligned}
\nabla_{\theta\theta}\hat{Q}(\dot{\theta}) &= -\hat{G}'\hat{\Omega}(\dot{\theta})^{-1}\hat{G} + 4\dot{\theta}n_X^{-1}\hat{\sigma}_V^2\hat{G}'\hat{\Omega}_0\hat{\Omega}(\dot{\theta})^{-2}\hat{g}(\dot{\theta}) + n_X^{-1}\hat{\sigma}_V^2\hat{g}(\dot{\theta})'\hat{\Omega}_0\hat{\Omega}(\dot{\theta})^{-2}\hat{g}(\dot{\theta}) \\
&\quad -4\dot{\theta}^2 n_X^{-2}\hat{\sigma}_V^4\hat{g}(\dot{\theta})'\hat{\Omega}_0^2\hat{\Omega}(\dot{\theta})^{-3}\hat{g}(\dot{\theta}) \\
&= -G'\Omega^{-1}G - (\hat{G}-G)\hat{\Omega}(\dot{\theta})^{-1}\hat{G} - G'(\hat{\Omega}(\dot{\theta})^{-1} - \Omega^{-1})\hat{G} - G'\hat{\Omega}(\dot{\theta})^{-1}(\hat{G}-G) + o_P(n) \\
&= -G'\Omega^{-1}G + o_P(n)
\end{aligned}$$

where the second equality follows by Lemmas 3(iv), 4(iii), 6(ii), 7(v), 8(iii) and M, and the third equality follows by Lemmas 4, 7(v), and 7(vi). □

Lemma 10. As $n,p\to\infty$, **(i)** $G'\Omega^{-1}G = \Theta(n)$; **(ii)** $(G'\Omega^{-1}G)^{-\frac{1}{2}}\nabla_\theta\hat{Q}(\theta_0) \xrightarrow{D} N(0,I_r)$; **(iii)** $(G'\Omega^{-1}G)^{-1}\nabla_{\theta\theta}\hat{Q}(\dot{\theta}) \xrightarrow{P} -I_r$.

Proof. For Part (i), note that $G'\Omega^{-1}G = G_0'\Omega_0^{-1}G_0 \cdot (n_Y^{-1}\sigma_\epsilon^2 + \theta_0^2 n_X^{-1}\sigma_V^2)^{-1} = \Theta(n)$ since $G_0 = \Lambda'\delta_X$ and $G_0'\Omega_0^{-1}G = O(1)$. For Part (ii), note that $G_0'\Omega_0^{-1}G_0$ is invertible by assumption, and by Part (i), $(G'\Omega^{-1}G)^{-\frac{1}{2}} = \Theta(n^{-\frac{1}{2}})$. Since $\hat{g}(\theta_0)\sim N(0,\Omega)$ by Equation (S.1) and Assumption 2, we have that $G'\Omega^{-1}\hat{g}(\theta_0)\sim N(0, G'\Omega^{-1}G)$, so that $(G'\Omega^{-1}G)^{-\frac{1}{2}}\nabla_\theta\hat{Q}(\theta_0) = -(G'\Omega^{-1}G)^{-\frac{1}{2}}G'\Omega^{-1}\hat{g}(\theta_0) + o_P(1) \xrightarrow{D} N(0,I_r)$. Part (iii) follows by $(G'\Omega^{-1}G)^{-1}\nabla_{\theta\theta}\hat{Q}(\dot{\theta}) = -I_r + o_P(1)$ by Part (i) and Lemma 9(ii). □

Web Appendix C.2 Proof of Theorem 2

Let $\Delta_{GG} = H^{-1}\Omega_0 H^{-1\prime} n_X^{-1}\sigma_V^2$ and $\Delta_G = \Delta_{GG}\theta_0$.

Lemma 11. As $n,p\to\infty$, **(i)** $\hat{\Delta}_{GG} - \Delta_{GG} = o_P(n^{-1}p)$; **(ii)** $\hat{\Delta}_G - \Delta_G = o_P(n^{-1}p)$.

Proof. We can write $\hat{\Delta}_{GG} - \Delta_{GG} = (\hat{\Omega}_0 - H^{-1}\Omega_0 H^{-1\prime})n_X^{-1}\hat{\sigma}_V^2 + H^{-1}\Omega_0 H^{-1\prime}n_X^{-1}(\hat{\sigma}_V^2 - \sigma_V^2)$, where first term on the RHS is $o_P(n^{-1}p)$ by Lemmas 3(iii), 6(ii), and M, and the second term on the RHS is $o_P(n^{-1}p)$ by Lemmas 1(ii), 3(i), and 6(ii). The result for Part (ii) directly follows since $\hat{\Delta}_G - \Delta_G = (\hat{\Delta}_{GG} - \Delta_{GG})\theta_0$. □

Let $\bar{S}_0 = \Omega^{-\frac{1}{2}}g(\theta_0)$ and $\bar{T}_0 = (\Delta_{GG} - \Delta_G\Omega^{-1}\Delta_G)^{-\frac{1}{2}}(\bar{G} - \Delta_G\Omega^{-1}g(\theta_0))$. Under our weak instrument asymptotics, $\Lambda'\delta_X = \Theta(n^{-\frac{1}{2}}p^{\frac{1}{2}})$, so that $G = -H^{-1}\Lambda'\delta_X = O_P(n^{-\frac{1}{2}}p^{\frac{1}{2}})$ by Lemma 1(ii).

By Assumption 1, under $H_0:\theta=\theta_0$,

$$\begin{bmatrix} g(\theta_0) \\ \bar{G} - \Delta_G\Omega^{-1}g(\theta_0)\end{bmatrix} \sim N\left(\begin{bmatrix}0\\G\end{bmatrix}, \begin{bmatrix}\Omega & 0 \\ 0 & \Delta_{GG} - \Delta_G\Omega^{-1}\Delta_G\end{bmatrix}\right).$$

Let $\bar{S}_0 = \Omega^{-\frac{1}{2}}g(\theta_0)$ and $\bar{T}_0 = (\Omega_X - \Delta_G\Omega^{-1}\Delta_G)^{-\frac{1}{2}}(\bar{G} - \Delta_G\Omega^{-1}g(\theta_0))$. By the above, $\bar{S}_0$ and $\bar{T}_0$ are jointly normal and uncorrelated, and hence are independent statistics. Let $\bar{Q}_{S,0} = \bar{S}_0'\bar{S}_0$, $\bar{Q}_{ST,0} = \bar{S}_0'\bar{T}_0$, and $\bar{Q}_{T,0} = \bar{T}_0'\bar{T}_0$. By identical arguments used in Smith (2007, Proof of Theorem 3.2, p.252), conditional on $\mathcal{Z}_T \sim N\big((\Delta_{GG} - \Delta_G\Omega^{-1}\Delta_G)^{-\frac{1}{2}}G, I_r\big)$, we have that $\bar{Q}_{S,0} \xrightarrow{D} \chi^2(r)$,

$\bar{Q}_{T,0}^{-1}\bar{Q}_{ST,0}^2 \xrightarrow{D} \chi^2(1)$, and $\big(\bar{Q}_{S,0}-\bar{Q}_{T,0}+\sqrt{(\bar{Q}_{S,0}-\bar{Q}_{T,0})^2+4\bar{Q}_{ST,0}^2}\big)/2 \xrightarrow{D} \big(\chi^2(1)+\chi^2(r-1)-\mathcal{Z}_T'\mathcal{Z}_T+\sqrt{(\chi^2(1)+\chi^2(r-1)-\mathcal{Z}_T'\mathcal{Z}_T)^2+4\chi^2(1)(\mathcal{Z}_T'\mathcal{Z}_T)}\big)/2$. We are left to show that $\bar{Q}_S=\bar{Q}_{S,0}+o_P(1)$, $\bar{Q}_{ST}=\bar{Q}_{ST,0}+o_P(1)$, and $\bar{Q}_T=\bar{Q}_{T,0}+o_P(1)$, so that the result of the theorem follows by Slutsky's lemma.

First, note that $(\hat{G}-\hat{\Delta}_G\hat{\Omega}(\theta_0)^{-1}\hat{g}(\theta_0))-(\bar{G}-\Delta_G\Omega^{-1}g(\theta_0))=(\hat{G}-\bar{G})-(\hat{\Delta}_G-\Delta_G)\hat{\Omega}(\theta_0)^{-1}\hat{g}(\theta_0)-\Delta_G(\hat{\Omega}(\theta_0)^{-1}-\Omega^{-1})\hat{g}(\theta_0)-\Delta_G\Omega^{-1}(\hat{g}(\theta_0)-g(\theta_0))=(\hat{G}-\bar{G})+o_P(n^{-\frac{1}{2}}p^{\frac{1}{2}})$, by Lemmas 1(i), 3(i), 6(ii), 7(ii), 7(v), 7(vi), 8(i), 8(ii), 11(ii), and M.

Using the expansion of $\hat{\lambda}_k-H^{-1}\lambda_k$, $k\in[p]$ from Equation (S.1), we can write

$$\begin{aligned}\hat{G}-\bar{G} &= -\sum_{k=1}^{p}(\hat{\lambda}_k-H^{-1}\lambda_k)\delta_{X_k}-\sum_{k=1}^{p}(\hat{\lambda}_k-H^{-1}\lambda_k)\varepsilon_{X_k}\\ &= H'\frac{1}{n_Z}\sum_{i=1}^{n_Z}\sum_{k=1}^{p}F_ie_{ki}\delta_{X_k}-\Big(\frac{1}{n_Z}\sum_{i=1}^{n_Z}\hat{F}_i(F_i-H^{-1\prime}\hat{F}_i)'\Big)(\Lambda'\delta_X)\\ &\quad -\frac{1}{n_Z}\sum_{i=1}^{n_Z}(\hat{F}_i-H'F_i)\big(\sum_{k=1}^{p}e_{ki}\delta_{X_k}\big)-\sum_{k=1}^{p}(\hat{\lambda}_k-H^{-1}\lambda_k)\varepsilon_{X_k}\\ &:= \mathcal{R}_4+\mathcal{R}_5+\mathcal{R}_6+\mathcal{R}_7.\end{aligned}$$

By CS, $\|\mathcal{R}_4\|\le\|H\|\cdot C_1n^{-a}n_Z^{-\frac{1}{2}}\|(n_Zp)^{-\frac{1}{2}}\sum_{i=1}^{n_Z}\sum_{k=1}^{p}F_ie_{ki}\|=O_P(n^{-(\frac{1}{2}+a)})=o_P(n^{-\frac{1}{2}}p^{\frac{1}{2}})$ for some constant C_1, by Lemma 1(i), $\delta_{X_k}=O(n^{-a}p^{-\frac{1}{2}})$ for all $k\in[p]$, and Assumption S0(iv). For $\mathcal{R}_5$, note that $\|\mathcal{R}_5\|\le\|H^{-1}\|\cdot\|n_Z^{-1}\hat{F}'(FH-\hat{F})\|\cdot\|\Lambda'\delta_X\|=O_P(\min(n,p)^{-1})O_P(n^{-\frac{1}{2}}p^{\frac{1}{2}})=o_P(n^{-\frac{1}{2}}p^{\frac{1}{2}})$ by Lemma 1(ii), and Lemma B.3 of Bai (2003, p.165), $\Lambda'\delta_X=\Theta(n^{-\frac{1}{2}}p^{\frac{1}{2}})$, and CS. Similarly, $\|\mathcal{R}_6\|\le C_1n^{-a}\big(n_Z^{-1}\sum_{i=1}^{n_Z}\|\hat{F}_i-H'F_i\|^2\big)^{\frac{1}{2}}\big(n_Z^{-1}\sum_{i=1}^{n_Z}|p^{-\frac{1}{2}}\sum_{k=1}^{p}e_{ki}|^2\big)^{\frac{1}{2}}=O_P(n^{-a}\min(n,p)^{-\frac{1}{2}})=o_P(n^{-\frac{1}{2}}p^{\frac{1}{2}})$ for some constant C_1, where the first equality follows by $\delta_{X_k}=O(n^{-a}p^{-\frac{1}{2}})$ for all $k\in[p]$, Lemma A.1 of Bai (2003, p.159), Assumption S0(iv), M, and CS. The second equality follows the rate restriction $pn^{-\frac{1}{2}}\to\infty$, as $n,p\to\infty$. Finally, $\|\mathcal{R}_7\|=o_P(n^{-\frac{1}{2}}p^{\frac{1}{2}})$ by identical arguments used in Proof of Lemma 8(i). Hence, $(\hat{G}-\hat{\Delta}_G\hat{\Omega}(\theta_0)^{-1}\hat{g}(\theta_0))-(\bar{G}-\Delta_G\Omega^{-1}g(\theta_0))=o_P(n^{-\frac{1}{2}}p^{\frac{1}{2}})$.

Also, $(\hat{\Delta}_{GG}-\hat{\Delta}_G\hat{\Omega}(\theta_0)^{-1}\hat{\Delta}_G)-(\Delta_{GG}-\Delta_G\Omega^{-1}\Delta_G)=(\hat{\Delta}_{GG}-\Delta_{GG})-(\hat{\Delta}_G-\Delta_G)\hat{\Omega}(\theta_0)^{-1}\hat{\Delta}_G-\Delta_G(\hat{\Omega}(\theta_0)^{-1}-\Omega^{-1})\hat{\Delta}_G-\Delta_G\Omega^{-1}(\hat{\Delta}_G-\Delta_G)=o_P(n^{-1}p)$ by Lemmas 1(ii), 3(i), 7(ii), 7(v), 7(vi), 11, and M. Then, by similar arguments used in the proof of Lemmas 7(v) and 7(vi), we have that $(\hat{\Delta}_{GG}-\hat{\Delta}_G\hat{\Omega}(\theta_0)^{-1}\hat{\Delta}_G)^{-1}=O_P(np^{-1})$, $(\Delta_{GG}-\Delta_G\Omega^{-1}\Delta_G)^{-1}=O_P(np^{-1})$, and $(\hat{\Delta}_{GG}-\hat{\Delta}_G\hat{\Omega}(\theta_0)^{-1}\hat{\Delta}_G)^{-1}-(\Delta_{GG}-\Delta_G\Omega^{-1}\Delta_G)^{-1}=o_P(np^{-1})$. Now, note that $\bar{T}-\bar{T}_0=\big((\hat{\Delta}_{GG}-\hat{\Delta}_G\hat{\Omega}(\theta_0)^{-1}\hat{\Delta}_G)^{-\frac{1}{2}}-(\Delta_{GG}-\Delta_G\Omega^{-1}\Delta_G)^{-\frac{1}{2}}\big)(\hat{G}-\hat{\Delta}_G\hat{\Omega}(\theta_0)^{-1}\hat{g}(\theta_0))+(\Delta_{GG}-\Delta_G\Omega^{-1}\Delta_G)^{-\frac{1}{2}}\big((\hat{G}-\hat{\Delta}_G\hat{\Omega}(\theta_0)^{-1}\hat{g}(\theta_0))-(\bar{G}-\Delta_G\Omega^{-1}g(\theta_0))\big)$. Therefore, $\bar{T}-\bar{T}_0=o_P(1)$.

Next, $\bar{S}-\bar{S}_0=(\hat{\Omega}(\theta_0)^{-\frac{1}{2}}-\Omega^{-\frac{1}{2}})\hat{g}(\theta_0)+\Omega^{-\frac{1}{2}}(\hat{g}(\theta_0)-g(\theta_0))=o_P(1)$ by Lemmas 7(v), 7(vi), 8(i), and 8(ii).

Having established the consistency of $\bar{S}$ for $\bar{S}_0$, and $\bar{T}$ for $\bar{T}_0$, we have that $\bar{Q}_S-\bar{Q}_{S,0}=(\bar{S}-\bar{S}_0)'(\bar{S}-\bar{S}_0)+\bar{S}_0'(\bar{S}-\bar{S}_0)+(\bar{S}-\bar{S}_0)'\bar{S}_0=o_P(1)$, $\bar{Q}_{ST}-\bar{Q}_{ST,0}=\bar{S}'\bar{T}-\bar{S}_0'\bar{T}_0=(\bar{S}-\bar{S}_0)'(\bar{T}-\bar{T}_0)+\bar{S}_0'(\bar{T}-\bar{T}_0)+(\bar{S}-\bar{S}_0)'\bar{T}_0=o_P(1)$, and $\bar{Q}_T-\bar{Q}_{T,0}=(\bar{T}-\bar{T}_0)'(\bar{T}-\bar{T}_0)+\bar{T}_0'(\bar{T}-\bar{T}_0)+(\bar{T}-\bar{T}_0)'\bar{T}_0=o_P(1)$. □

Web Appendix C.3 Proof of Theorem 3

Let $\hat{g}_S(\theta) = \Gamma'_S\hat{g}(\theta)$, $\hat{G}_S = \Gamma'_S\hat{G}$, $\hat{\Omega}_S(\theta) = \Gamma'_S\hat{\Omega}(\theta)\Gamma_S$, $g_S(\theta) = \Gamma'_S g(\theta)$, $G_S = \Gamma'_S G$, $\Omega_S(\theta) = \Gamma'_S\Omega(\theta)\Gamma_S$, $\Omega_{\mathcal{S}} = \Omega_{\mathcal{S}}(\theta_0)$, and D the $p \times p$ diagonal matrix with its (j,j)-th element given by $(\Delta_{GG})_{jj}$.

We work with the joint normality model

$$\begin{bmatrix} \hat{\theta}_{\mathcal{S}} \\ \hat{\mathcal{T}} \end{bmatrix} \sim N\left(\begin{bmatrix} \theta_0 \\ D^{-\frac{1}{2}}G \end{bmatrix}, \begin{bmatrix} V_{\mathcal{S}} & C'_G \\ C_G & V_G \end{bmatrix}\right),$$

where $V_S = (G'_S\Omega_S G_S)^{-1}$, $C_G = -D^{-\frac{1}{2}}\Delta_{GG}\Gamma_{\mathcal{S}}\Omega_{\mathcal{S}}^{-1}G_{\mathcal{S}}V_{\mathcal{S}}\theta_0$, and $V_G = D^{-\frac{1}{2}}\Delta_{GG}D^{-\frac{1}{2}}$.

To remove dependence of $\hat{\theta}_S$ on $D^{-\frac{1}{2}}G$, we condition on a sufficient statistic for the unknown nuisance parameter $D^{-\frac{1}{2}}G$, which is given by $U = \hat{\mathcal{T}} - C_G V_{\mathcal{S}}^{-1}\hat{\theta}_{\mathcal{S}}$. Note that $\hat{\theta}_{\mathcal{S}}$ and U are uncorrelated. Moreover, conditional on $U = u$, and under $\mathcal{H}_0 : \theta = \theta_0$, we have that $\hat{\mathcal{T}} = u + C_G V_{\mathcal{S}}^{-1}\hat{\theta}_{\mathcal{S}}$, which is a random variable with distribution $u + C_G V_{\mathcal{S}}^{-1}(\theta_0 + V_{\mathcal{S}}^{\frac{1}{2}}\mathcal{K})$ where $\mathcal{K} \sim N(0,1)$. Thus, if $\mathcal{S}$ is the selection event, and $\mathcal{R}$ is the set of indices of the estimated factor loadings corresponding to the retained instruments, under $\mathcal{H}_0 : \theta = \theta_0$,

$$\begin{aligned} P(\hat{\theta}_{\mathcal{S}} \leq w|\mathcal{S}, U = u) &= \frac{P(\{\hat{\theta}_{\mathcal{S}} \leq w\} \cap \mathcal{S}|U = u)}{P(\mathcal{S}|U = u)} \\ &= \frac{P\big(\{\theta_0 + V_{\mathcal{S}}^{\frac{1}{2}}\mathcal{K} \leq w\} \cap \{|(\bar{u})_j| > c_\delta, j \in \mathcal{R}\} \cap \{|(\bar{u})_k| \leq c_\delta, k \in [r]\backslash\mathcal{R}\}\big)}{P\big(\{|(\bar{u})_j| > c_\delta, j \in \mathcal{R}\} \cap \{|(\bar{u})_k| \leq c_\delta, k \in [r]\backslash\mathcal{R}\}\big)}, \end{aligned}$$

where $\bar{u} = u + C_G V_{\mathcal{S}}^{-1}(\theta_0 + V_{\mathcal{S}}^{\frac{1}{2}}\mathcal{K})$, and $\mathcal{K} \sim N(0,1)$. □

The joint normality assumption with variance components V_S, C_G, and V_G are motivated by the following arguments. By Proof of Theorem 1, we have that $\hat{\theta}_{\mathcal{S}} - \theta_0 \approx -V_{\mathcal{S}}G'_{\mathcal{S}}\Omega_{\mathcal{S}}^{-1}\Gamma'_{\mathcal{S}}H^{-1}\Lambda'(\hat{\delta}_Y - \hat{\delta}_X\theta_0)$. By Proof of Lemma 4(ii), we have that $D^{-\frac{1}{2}}\bar{G} - D^{-\frac{1}{2}}G \approx -H^{-1}\Lambda'(\hat{\delta}_X - \delta_X)$. Under Assumption 2, we have that $Cov(\Lambda'(\hat{\delta}_Y - \hat{\delta}_X\theta_0), \Lambda'(\hat{\delta}_X - \delta_X)) = -\Omega_0 n_X^{-1}\sigma_V^2$, which motivates the expression for C_G. Also, $Var(\Lambda'(\hat{\delta}_X - \delta_X)) = \Omega_0 n_X^{-1}\sigma_V^2$, which motivates the expression for V_G.

To make the result of Theorem 3 operational, we take the empirical distributions for probabilities in the numerator and denominator on the RHS of the expression for $P(\hat{\theta}_{\mathcal{S}} \leq w|\mathcal{S}, U = u)$ above, based on draws of $\mathcal{K} \sim N(0,1)$. Also, we replace the variance components $V_{\mathcal{S}}$, C_G and V_G with estimates $\hat{V}_{\mathcal{S}} = (\hat{G}'_S\hat{\Omega}(\hat{\theta}_{\mathcal{S}})^{-1}\hat{G}_{\mathcal{S}})^{-1}$, $\hat{C}_G = -\hat{D}^{-\frac{1}{2}}\hat{\Delta}_{GG}\Gamma_{\mathcal{S}}\hat{\Omega}_{\mathcal{S}}(\hat{\theta}_{\mathcal{S}})^{-1}\hat{G}_{\mathcal{S}}\hat{V}_{\mathcal{S}}\hat{\theta}_{\mathcal{S}}$, and $\hat{V}_G = \hat{D}^{-\frac{1}{2}}\hat{\Delta}_{GG}\hat{D}^{-\frac{1}{2}}$, where $\hat{D}$ is a $p \times p$ diagonal matrix with (j,j)-th element equal to $(\hat{\Delta}_{GG})_{jj}$. We note that the errors from estimating the variance components $V_{\mathcal{S}}$ and C_G, and estimation error $\hat{\mathcal{T}} - D^{-\frac{1}{2}}\bar{G}$, are not ignorable. Therefore, the joint normality approximation used above is not exact.

Web Appendix D Further simulation results

In this section, we present additional simulation results from the models discussed in Section 4. We briefly describe the alternative summary data methods that we used. The "CLR" test is the conditional likelihood ratio test proposed in Wang and Kang (2021). The "DecorrIVW" test is the de-correlated IVW test when variants are pruned at $R^2 \leq 0.1$ unless otherwise stated. The "RAPS" test is the robust adjusted profile score test proposed in Zhao et al., 2020 under a robust loss function with no overdispersion parameter. The "DIVW" test is the de-biased IVW test proposed in Ye et al. (2021). The "RAD" test is a radial IVW regression approach with second-order weights proposed in Bowden et al. (2019).

For indicating the level of pruning used, the tests CLR-0, CLR-1, CLR-2, and CLR-4 will denote the CLR test pruned to near-independence after selecting the strongest associated variant with LDL-C ($R^2 \leq 0.01$), $R^2 \leq 0.1$, $R^2 \leq 0.2$, and $R^2 \leq 0.4$, respectively. Likewise, RAPS-0, RAPS-1, and other tests are defined analogously.

For the power plots in Figures S1–S5, the first rows (Model 1) present the performance of tests under correct model specification and weak instruments. The second and third rows present the performance of tests when variants have local-to-zero direct effects τ on the outcome; $\delta_Y = \delta_X \theta_0 + n^{-\frac{1}{2}}\tau$. The second rows (Model 2) describe the setting where all variants have equal effects τ on the outcome, and the third rows (Model 3) describe the setting $\tau = \tau_2$, where τ_2 is a fixed effect, and each element of τ_2 is drawn from the uniform distribution $U(-1, 1)$. The fourth and fifth rows describe the setting where the variant correlation matrix available to the researcher $\bar{\rho}$ is different from the true variant correlation matrix ρ used to generate the summary genetic association data. For the fourth row (Model 4), we assume the correlation estimates available to the researcher satisfies $\bar{\rho}_{jk} = \rho_{jk} - \kappa_0$, for some $\kappa_0 > 0$, and in the fifth rows (Model 5), we assume the correlation estimates available to the researcher satisfies $\bar{\rho}_{jk} = sgn(\rho_{jk}) \cdot |\rho_{jk}|^{\kappa_2}$, for some $\kappa_2 > 0$.

In Figure S1, the results are presented for other methods not included in the plot of Figure 1 in the main text, for $p = 180$ variants. Figures S2–S3 present power results under $p = 90$ variants, and Figures S4–S5 present power results under $p = 360$ variants. Figure S6 presents histograms (under Model 1) of the standardized S-LIML estimates based on conventional standard errors, varying with the ratio $p/\sqrt{n_Z}$, where n_Z is the sample size used to generate an estimated variance-covariance matrix of variants $\widehat{var}(Z)$. The root mean square error (RMSE) of the S-LIML estimates is also indicated. Figure S7 presents histograms (under Model 1) of the standardized F-LIML estimates varying with the ratio $p/\sqrt{n_Z}$, and RMSE of methods which provide point estimates. We note that the RMSE of alternative summary data methods are not varying with $p/\sqrt{n_Z}$, since they did not require an estimate of $var(Z)$. Finally, Figure S8 presents the RMSE results of estimators under Model 3 (local-to-zero direct

variant effects on the outcome) when varying the number of estimated factors $\hat{r}$ away from the true number of factors $r = 11$. Figure S9 presents the power performance of the factor-based methods under misspecified correlation structure (Model 5) and for varied number of estimated factors $\widehat{r}$.

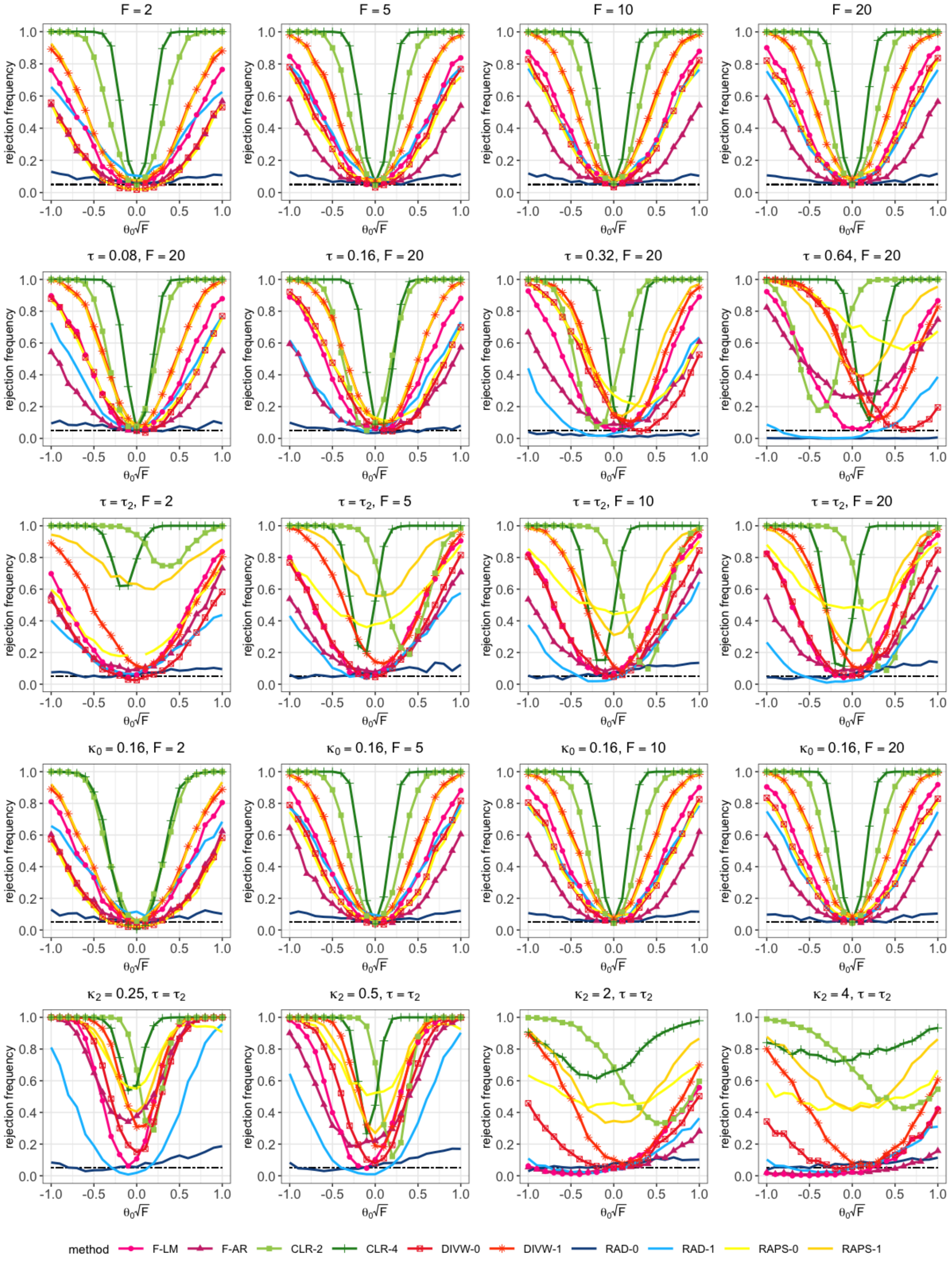

Figure S1. Power results when testing the null hypothesis $\mathcal{H}_0 : \theta_0 = 0$ for a 5% level test ($p = 180$ variants). The first row of panels correspond to Model 1 in Section 4.1. The second and third rows of panels

correspond to Models 2 and 3 in Section 4.2. The fourth and last rows of panels correspond to Models 4 and 5 in Section 4.3.

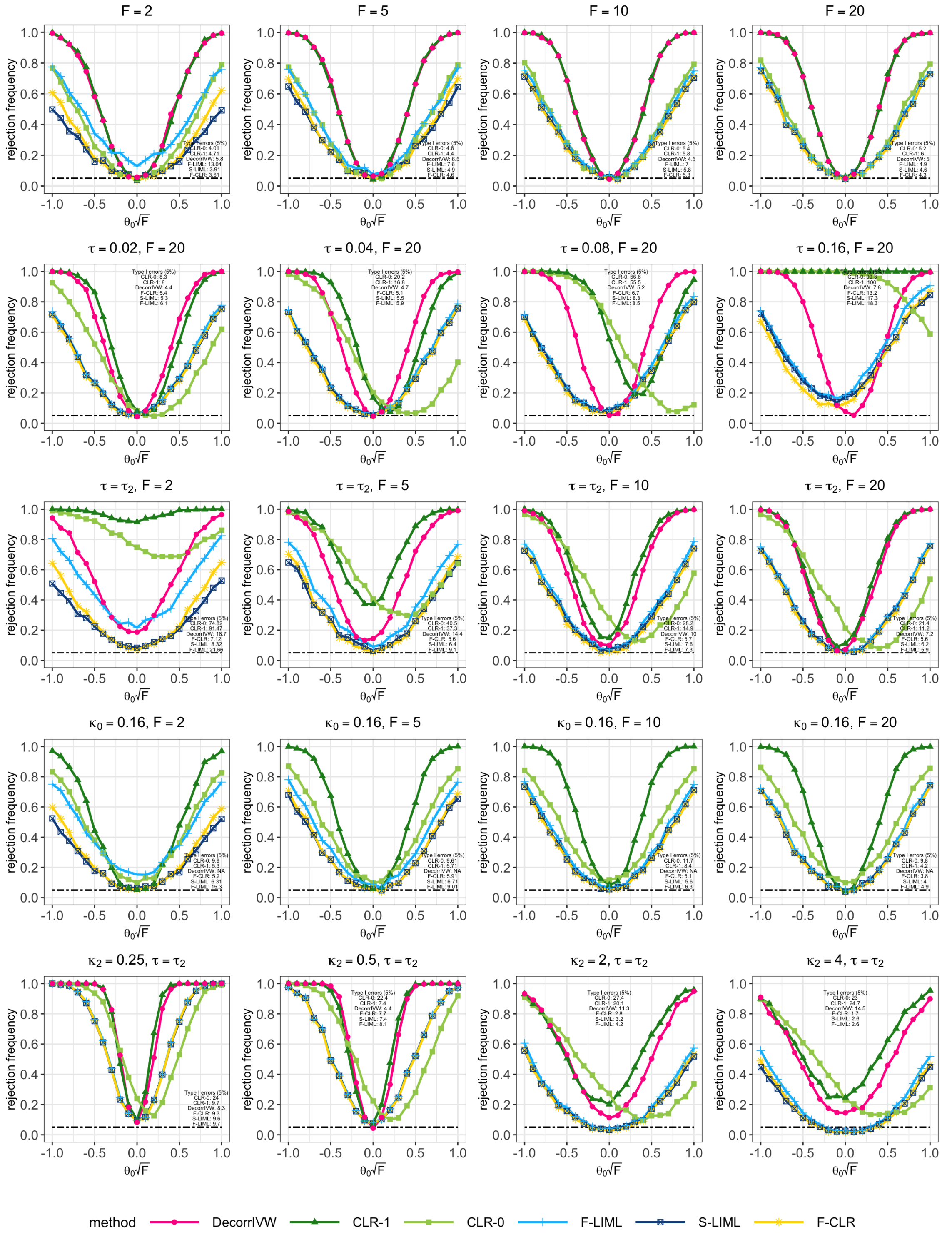

Figure S2. Power results when testing the null hypothesis $\mathcal{H}_0 : \theta_0 = 0$ for a 5% level test ($p = 90$ variants). The first row of panels correspond to Model 1 in Section 4.1. The second and third rows of panels correspond to Models 2 and 3 in Section 4.2. The fourth and last rows of panels correspond to Models 4 and 5 in Section 4.3.

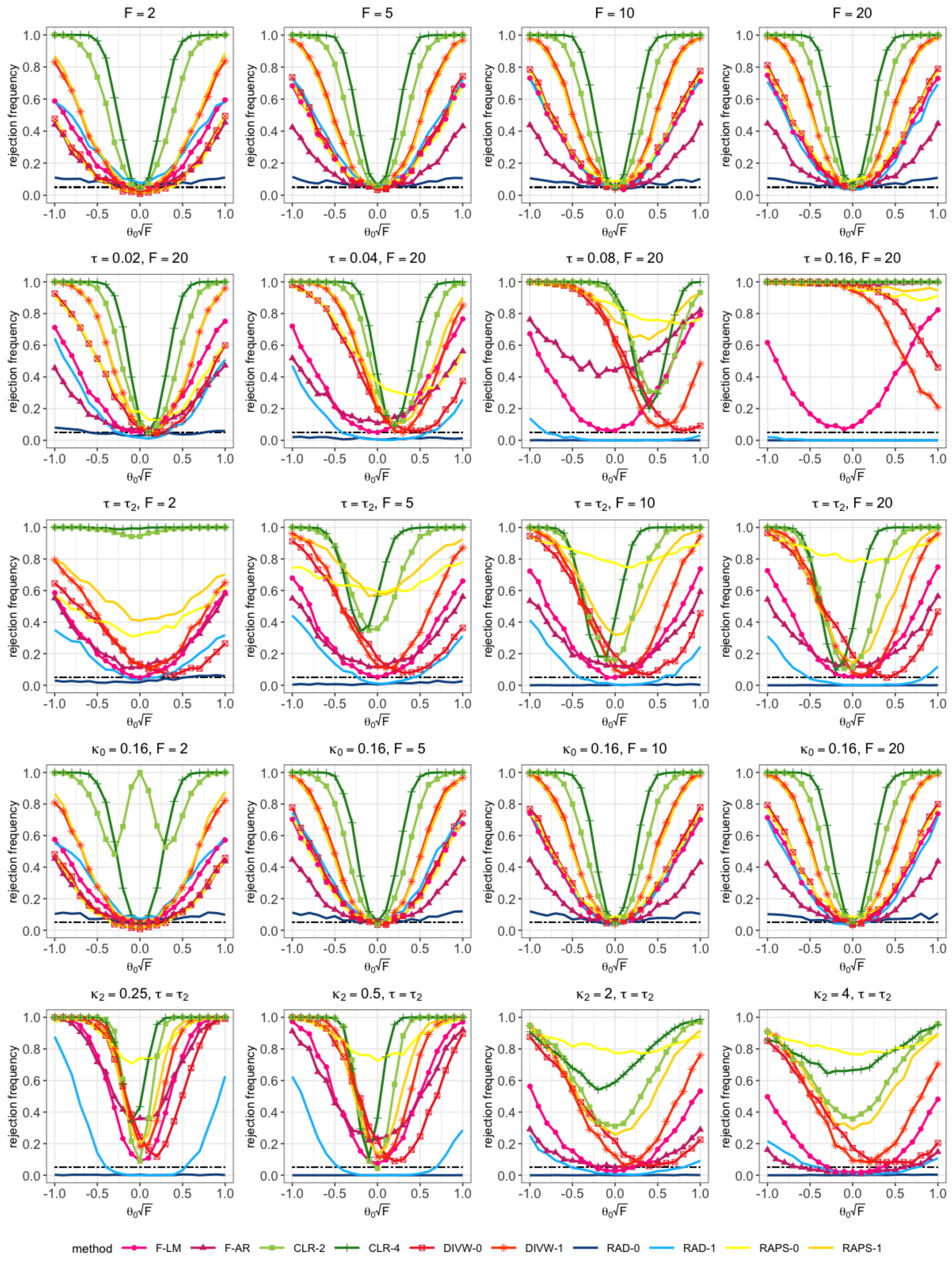

Figure S3. Power results when testing the null hypothesis $\mathcal{H}_0 : \theta_0 = 0$ for a 5% level test ($p = 90$ variants). The first row of panels correspond to Model 1 in Section 4.1. The second and third rows of panels correspond to Models 2 and 3 in Section 4.2. The fourth and last rows of panels correspond to Models 4 and 5 in Section 4.3.

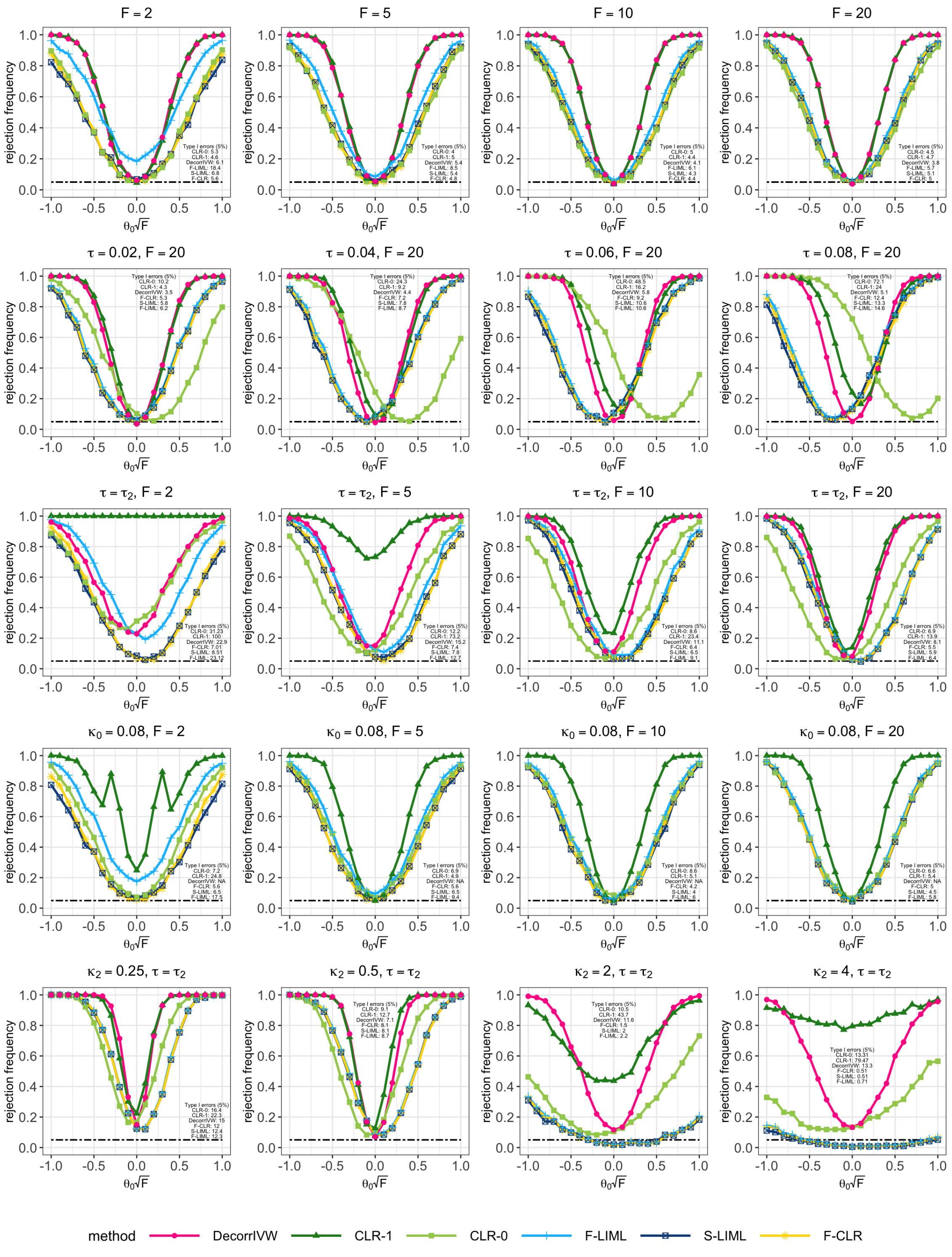

Figure S4. Power results when testing the null hypothesis $\mathcal{H}_0 : \theta_0 = 0$ for a 5% level test ($p = 360$ variants). The first row of panels correspond to Model 1 in Section 4.1. The second and third rows of panels correspond to Models 2 and 3 in Section 4.2. The fourth and last rows of panels correspond to Models 4 and 5 in Section 4.3.

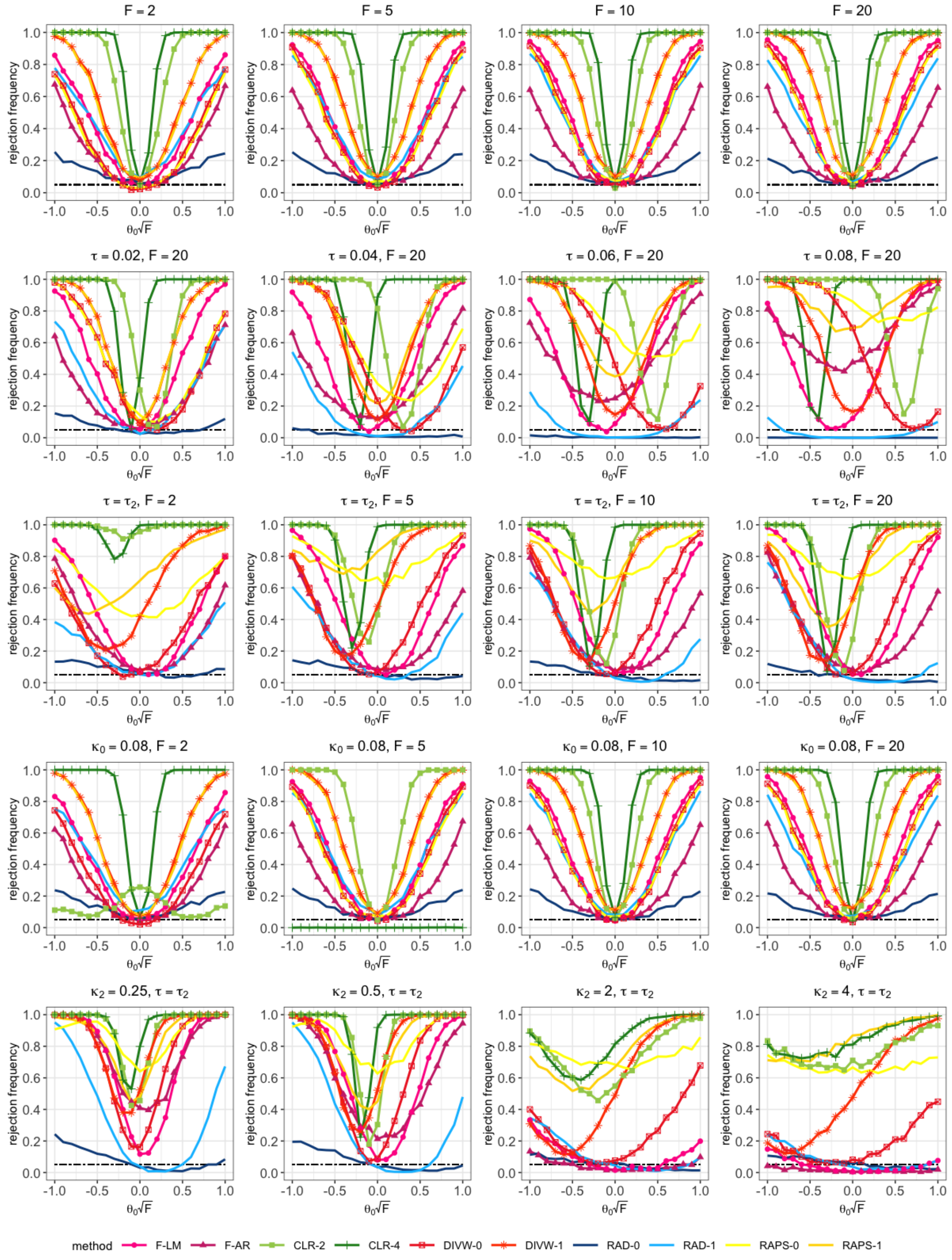

Figure S5. Power results when testing the null hypothesis $\mathcal{H}_0 : \theta_0 = 0$ for a 5% level test ($p = 360$ variants). The first row of panels correspond to Model 1 in Section 4.1. The second and third rows of panels correspond to Models 2 and 3 in Section 4.2. The fourth and last rows of panels correspond to Models 4 and 5 in Section 4.3.

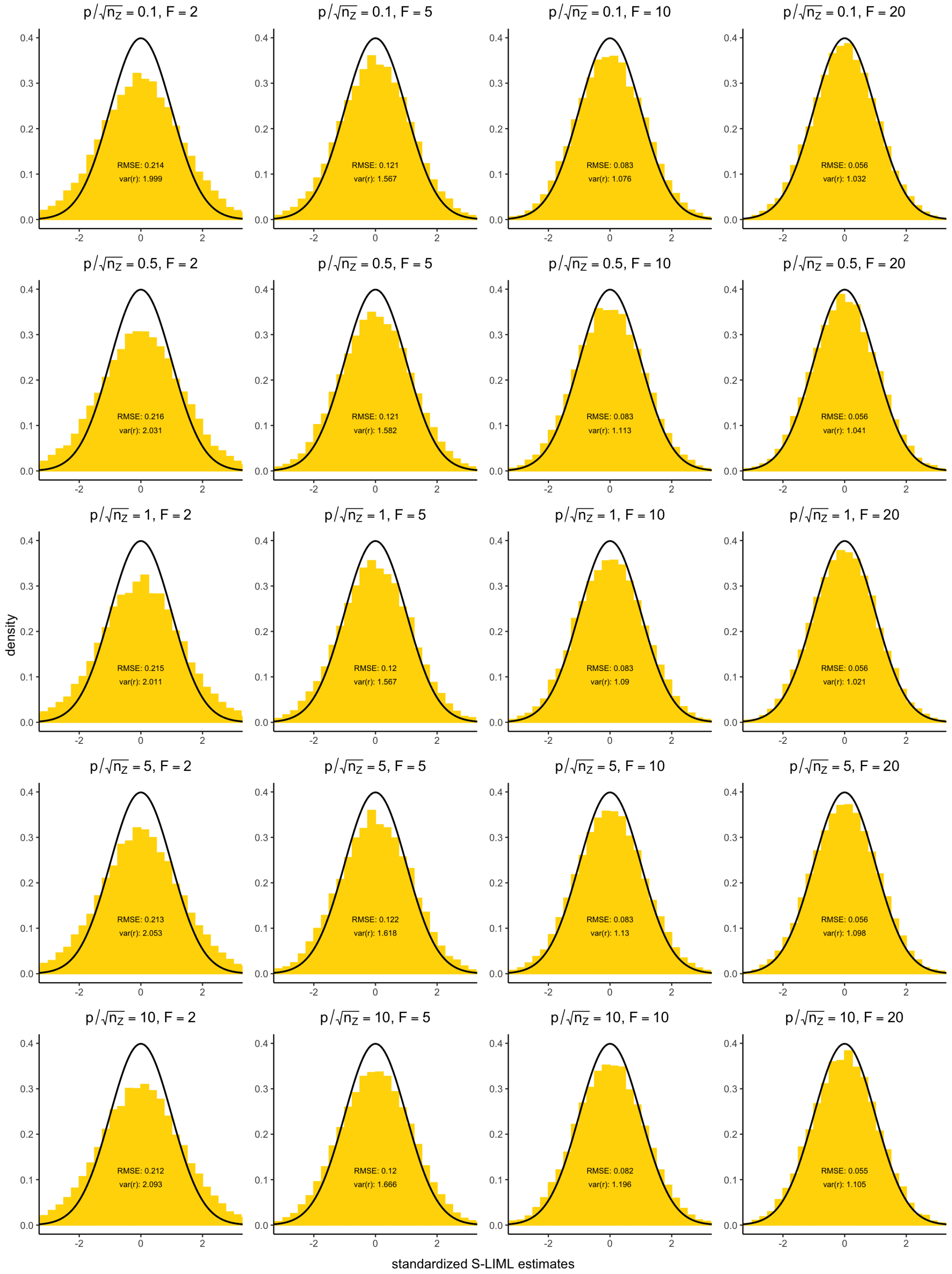

Figure S6. The performance of S-LIML under conventional standard errors for $p = 180$ (Model 1). Here, the $\sqrt{n_Z}$-order sampling error in estimates $\widehat{var}(Z_j)$, $j \in [p]$, is chosen to maintain a particular value of the ratio $p/\sqrt{n_Z}$, $var(r)$ notes the variance of the number of selected factors by S-LIML, and the solid black line is the $N(0,1)$ density curve.

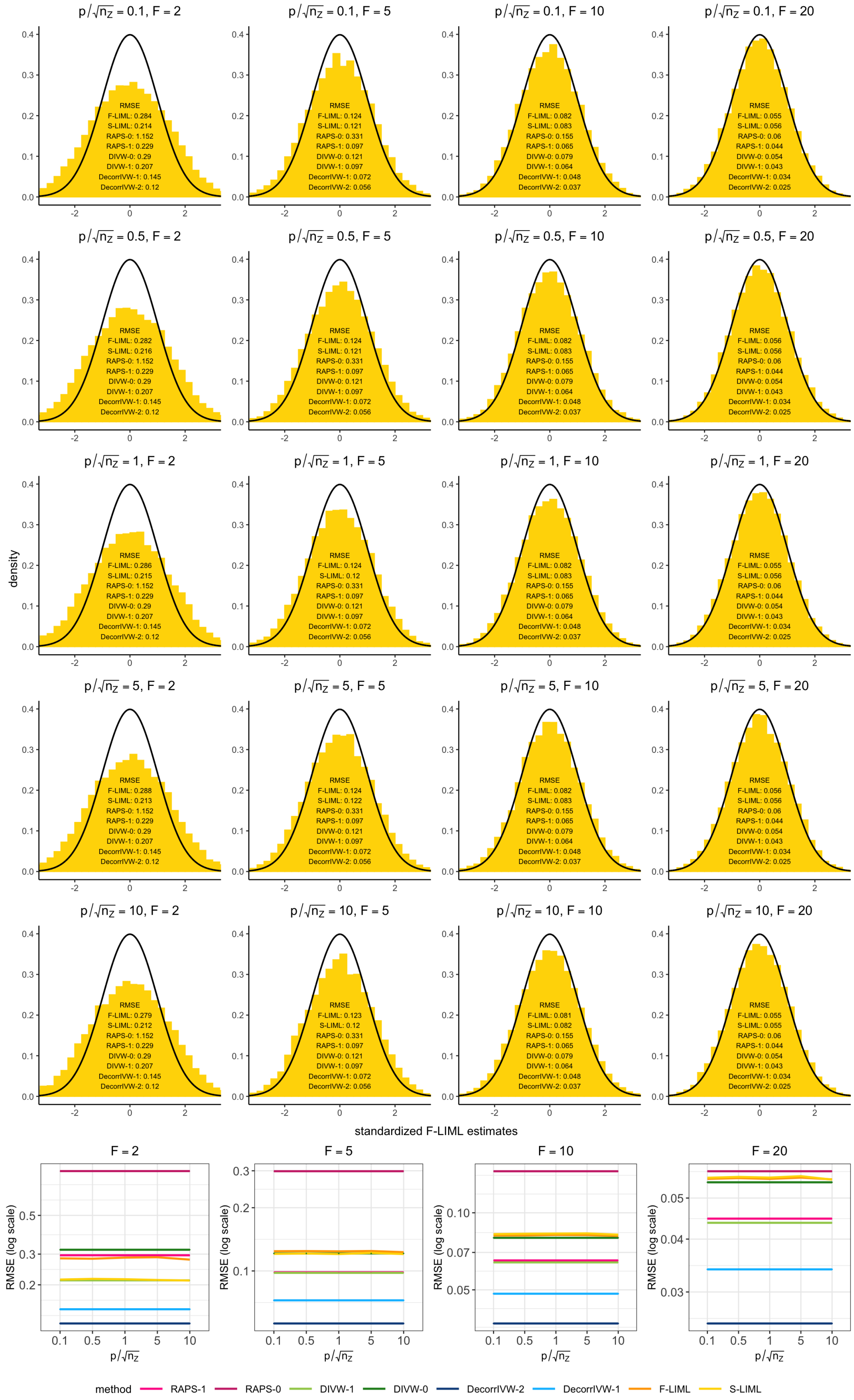

Figure S7. RMSE results under correct specification (Model 1) for $p = 180$. The histograms correspond to the standardized F-LIML estimates, and the sold black line is the $N(0, 1)$ density curve. Here, the $\sqrt{n_Z}$-order sampling error in estimates $\widehat{var}(Z_j)$, $j \in [p]$, is chosen to maintain a particular value of the ratio $p/\sqrt{n_Z}$.

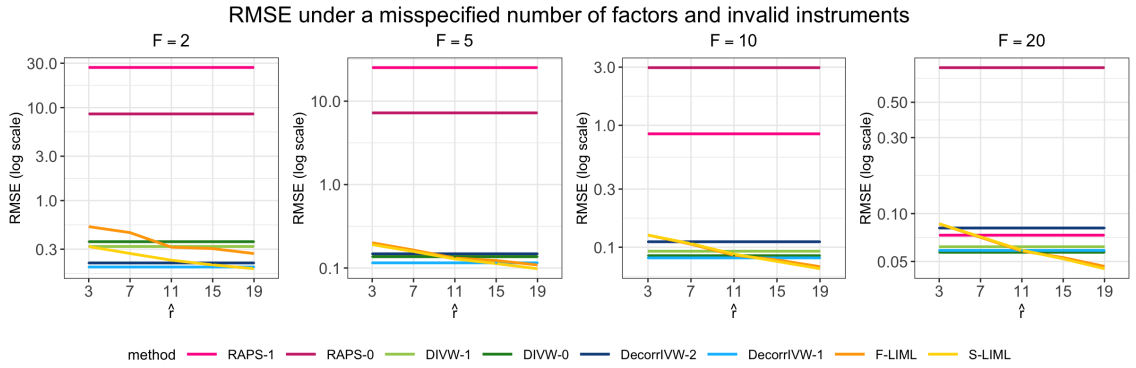

Figure S8. Root-mean squared error (RMSE) results under locally invalid instruments (Model 3) for $p = 180$ and misspecification $\hat{r} \neq r$. The true number of factors is $r = 11$.

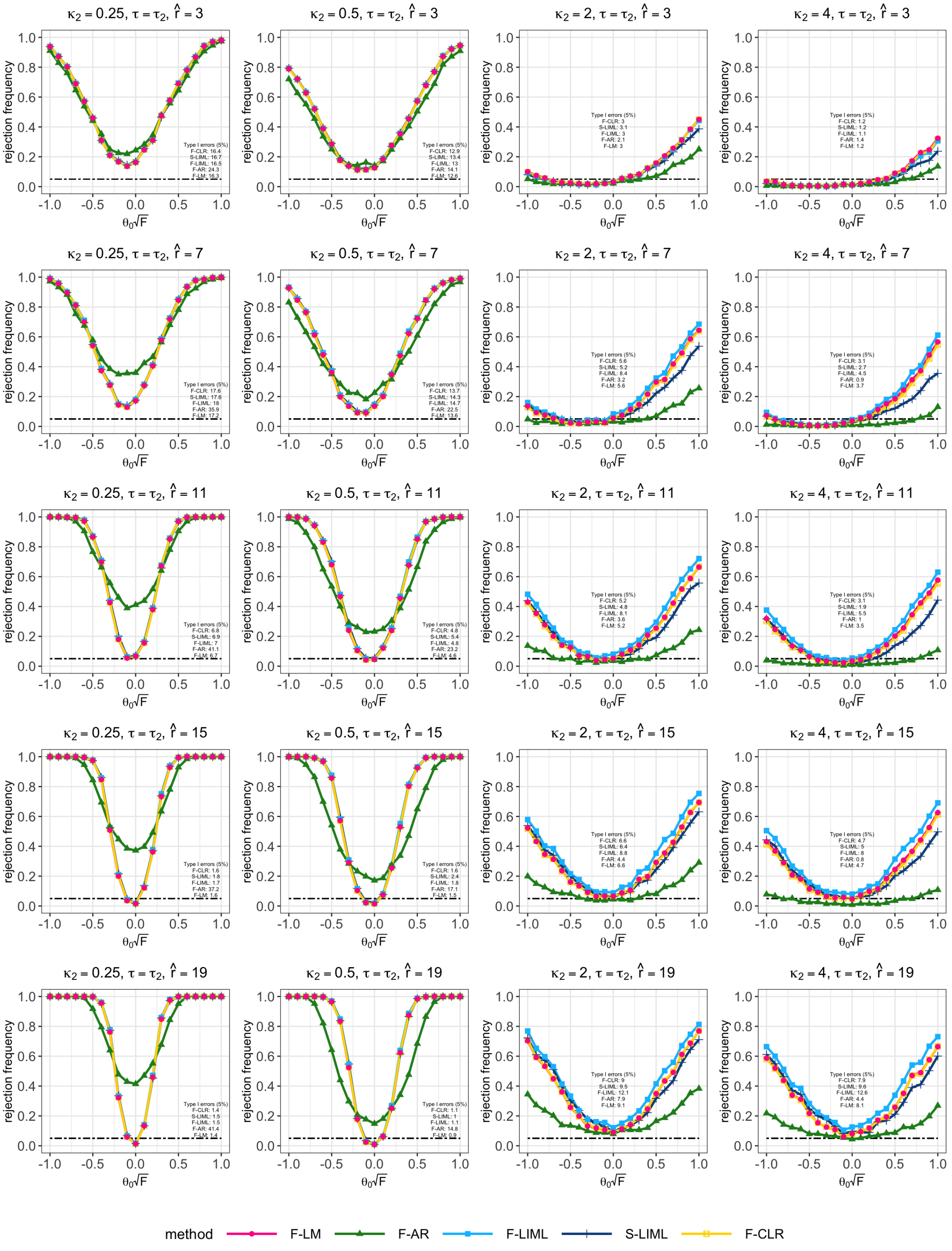

Figure S9. Power results when testing the null hypothesis $\mathcal{H}_0 : \theta_0 = 0$ for a 5% level test ($p = 180$ variants) under Model 5 in Section 4.3 and varied number of estimated factors $\widehat{r}$ selected as instruments.

Web Appendix D.1 S-LIML tests for non-zero values of the causal effect

Our inference strategy with S-LIML attempts to account for the first stage screening of relevant factors as instruments. There are, however, two cases where this may not be necessary; i.e. where we may be able to make correct inferences by ignoring the instrument selection step, and by simply using the usual GMM-based standard errors (as in Theorem 1). First, the procedure with S-LIML is more of a finite-sample correction and therefore for large enough sample sizes (and thus strong enough instruments) the correction may not have much of an influence. The second case is when we are interested only in testing the null hypothesis that there is no causal effect. However, if we are interested in obtaining confidence intervals for potentially non-zero effects (for example, by inverting the test), then ignoring the correction step may lead to relatively poor coverage under weak instruments.

To illustrate this, we simulated from the same "correct specification" setting in Model 1 described in Section 4.1, and we present the coverage results of confidence intervals with the correction step, denoted S-LIML, and without the correction step, denoted S-LIML (naive). For comparison, we also note the results of the F-LIML method (which does not screen out any weak instruments, and therefore uses the full set of genetic factors as instruments), and F-CLR (which is an identification-robust test, and so asymptotically guarantees correct coverage).

Figure S10 shows that when the instruments are strong enough, it is not necessary to account for the uncertainty in instrument selection based on their relevance. Moreover, even when the instruments are weak, and when the true causal effect is 0, there appears to be no coverage loss from ignoring the post-selection correction step. However, as the true causal effect moves away from 0, our inference strategy with S-LIML is able to achieve better coverage under weak instruments than if the uncertainty in instrument selection is ignored.

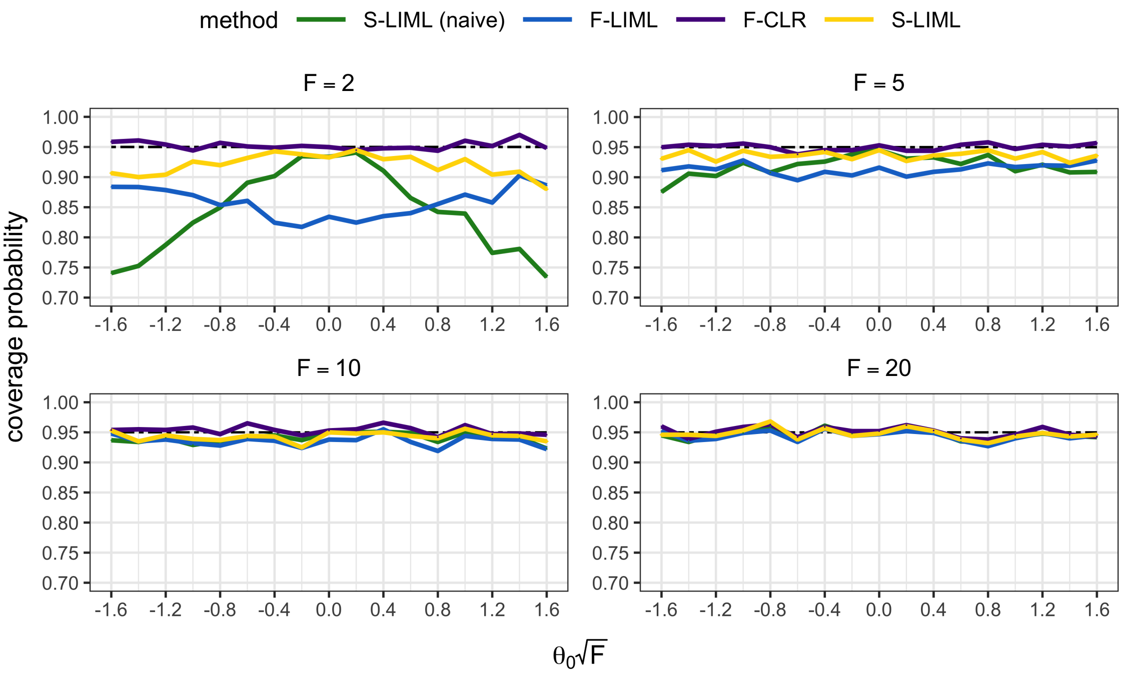

Figure S10. Coverage probabilities of confidence intervals under Model 1 and $\mathcal{H}_0 : \theta = \theta_0$ for varying θ_0. The dashed black line indicates nominal coverage was set to 0.95.

References

Bai, J. (2003). Inferential theory for factor models of large dimensions. *Econometrica 71*(1), 135–171.

Bai, J. and S. Ng (2002). Determining the number of factors in approximate factor models. *Econometrica 70*(1), 191–221.

Bai, J. and S. Ng (2010). Instrumental variable estimation in a data rich environment. *Econometric Theory 26*(6), 1577–1606.

Bowden, J., F. Del Greco M, C. Minelli, Q. Zhao, D. A. Lawlor, and N. A. Sheehan et al. (2019). Improving the accuracy of two-sample summary-data Mendelian randomization: Moving beyond the NOME assumption. *International Journal of Epidemiology 48*(3), 728–742.

Newey, W. K. and D. McFadden (1994). Large sample estimation and hypothesis testing. *Handbook of Econometrics 4*(1), 2111–2245.

Schooling, C. M., P. M. Lopez, Z. Yang, J. V. Zhao, S. L. Au Yeung, and J. V. Huang (2021). Use of multivariable Mendelian randomization to address biases due to competing risk before recruitment. *Frontiers in Genetics 11*(January), 1–10.

Smith, R. J. (2007). Weak instruments and empirical likelihood: a discussion of the papers by D.W.K. Andrews and J.H. Stock and Y. Kitamura. In *Advances in Economics and Econometrics,*

Theory and Applications: Ninth World Congress of the Econometric Society (Vol. 3 ed.)., pp. 238–260.

Wang, S. and H. Kang (2021). Weak-instrument robust tests in two-sample summary-data Mendelian randomization. *Biometrics (forthcoming)*, 1–15.

Ye, B. T., J. Shao, and H. Kang (2021). Debiased inverse-variance weighted estimator in two-sample summary-data Mendelian randomization. *Annals of Statistics 49*(4), 2079–2100.

Zhao, Q., J. Wang, G. Hemani, J. Bowden, and D. S. Small (2020). Statistical inference in two-sample summary-data Mendelian randomization using robust adjusted profile score. *Annals of Statistics 48*(3), 1742 – 1769.
